# Climate‐Associated Genetic Variation in Native and Introduced Populations of *Eustenopus villosus*, a Biological Control Agent for Yellow Starthistle

**DOI:** 10.1111/eva.70341

**Published:** 2026-11-05

**Authors:** Brittany S. Barker, Jessie A. Pelosi, Osman Sert, Katrina M. Dlugosch

**Affiliations:** ^1^ Oregon IPM Center Oregon State University Corvallis Oregon USA; ^2^ Department of Horticulture Oregon State University Corvallis Oregon USA; ^3^ Department of Ecology and Evolutionary Biology University of Arizona Tucson Arizona USA; ^4^ Department of Biology, Faculty of Science, Applied Biology Section Hacettepe University Ankara Türkiye

**Keywords:** *Centaurea solstitialis*, classical biological control, evolution, landscape genomics, local adaptation

## Abstract

Contemporary evolution in biological control systems is rarely studied, though biological agents are repeatedly exposed to novel environmental conditions upon introduction. Their ability to adapt to new conditions could influence management efficacy and reveal important aspects of environmental selection. Using a ddRADseq dataset, we tested for genomic evidence of adaptation to climate in populations of *Eustenopus villosus* (Boheman, Coleoptera: Curculionidae), the “hairy weevil”, which is used in classical biological control for yellow starthistle ( *Centaurea solstitialis* L., Asteraceae) in the western United States. We first estimated population structure in *E. villosus* across our samples of the native and introduced ranges and then used six complementary genotype‐environment association analyses to identify single nucleotide polymorphisms (SNPs) associated with climate in both native and introduced populations. Additionally, we used historical release records to categorize sampling sites according to their serial founding history and test for impacts of this history on total and putatively adaptive genomic variation in the introductions. We also tested whether candidate loci associated with climate in the native range exhibit similar associations in the introductions, or if new associations have evolved. We identified many candidate loci in both native and introduced populations and found little evidence of a decline in genomic variation, which suggests that serial founding events did not prevent climatic adaptation. Candidate loci differed between native and introduced populations, indicating that local adaptation has occurred in different regions of the genome after introduction of *E. villosus* . Our findings suggest an important role for novel contemporary adaptation in this biological control system and provide insight into how genetic variation can persist in biological control agents through introduction and serial founding events.

## 1 Introduction

Local adaptation of organisms to their biotic and abiotic environments is pervasive in nature, and is expected to play a key role in the distribution of species (Bridle et al. 2010; Sexton et al. 2009). Recently introduced species provide an outstanding opportunity to investigate the factors that limit distributions as they expand their ranges over new habitats (Sakai et al. 2001). In particular, classical biological control (i.e., biocontrol)—the importation of enemies from the native range of an introduced species to manage invading populations—can provide especially informative cases of species introductions, as the source, founding population size, and sites of introduction are often known precisely (Müller‐Schärer et al. 2020; Müller‐Schärer and Schaffner 2008; Yeates et al. 2012). A growing body of literature has demonstrated that evolution in biocontrol agents may occur on decadal timescales as they expand their range across new environments (Bean et al. 2012; Clark et al. 2022; McEvoy et al. 2012; Phillips et al. 2008; Szűcs et al. 2019).

Evolutionary considerations may also help to improve all stages of classical biocontrol programs, including pre‐release agent selection, establishment prediction, enhancing agent performance using artificial selection or genetic engineering, and post‐release environmental risk assessment (Hufbauer and Roderick 2005; Müller‐Schärer et al. 2020; Sentis et al. 2022; Wright and Bennett 2018). It may be particularly important to identify and quantify genetic variation associated with climatic adaptations in biocontrol agents, as the invasive species they are targeted to control may themselves occupy distributions outside their native climatic niches, or they are predicted to expand into new climates (Atwater et al. 2017; Riera et al. 2025). In general, however, the amounts, types, and sources of adaptive genetic variation that might affect the ability of introduced biocontrol agents to adapt to new environments are not well understood (Müller‐Schärer et al. 2020; Szűcs et al. 2019; Wright and Bennett 2018).

Biocontrol agents may lose genetic variation during the initial introduction and establishment process due to small founder sizes, which may result in reduced genetic diversity and changes in allele frequencies relative to the source population (Dlugosch, Anderson, et al. 2015; Roderick and Navajas 2003). Serial founder events may further reduce genetic diversity due to genetic drift, inbreeding, and population bottlenecks as agents expand their geographic range through a series of successive colonizations, which may limit adaptive potential and increase genetic load (Hagan et al. 2024; Peischl et al. 2013). Some studies have demonstrated evolution in life‐history traits of biocontrol insects that originated from a single source area as they expanded their range across new climates (Bean et al. 2012; Clark et al. 2022; McEvoy et al. 2012), which implies that single populations may retain sufficient genetic variation in traits affecting climatic adaptation (Dlugosch, Anderson, et al. 2015; Estoup et al. 2016). Additionally, there are examples of biocontrol agents originating from a single source area (one climate) that have come to occupy a wide range of climates in the introduced range (Hight et al. 1995; van Klinken et al. 2003), which suggests that climate mismatches did not prevent establishment success. However, others documented an increase in success rates in biocontrol agents in new climates after admixture between genetically distinct populations occurred, suggesting that adaptive genetic variation was potentially limited in those systems (Phillips et al. 2008; Szűcs et al. 2012), though admixture may not always be beneficial to colonizing populations (Barker et al. 2019).

In this study, we quantify genetic variation associated with climate in populations of *Eustenopus villosus* (Boheman) (Coleoptera: Curculionidae), common name “hairy weevil”, which is used in classical biocontrol programs for yellow starthistle [ *Centaurea solstitialis* L. (Asteraceae)] in the western United States. Native to Eurasia, *C. solstitialis* was introduced accidentally as a crop weed to California in the mid‐1800s, and has since aggressively invaded rangelands and natural areas across wide climatic gradients in the western United States (DiTomaso 1996; Gerlach et al. 1997). First released in the western United States in 1990, the *E. villosus* biocontrol population was reared from individuals collected from two geographically proximate sources near Thessaloniki, Greece (Figure 1) and underwent a series of successive dispersal events across the region (Fornasari et al. 1991; Pitcairn et al. 2008; Turner et al. 1994; Villegas 2000). The agent successfully established and became widespread across the western United States; however, populations have failed to control *C. solstitialis* (DiTomaso and Healy 2007). The success of *E. villosus* in terms of abundance appears to be influenced by local climate (Pitcairn et al. 2008; Swope and Satterthwaite 2012; Woodley et al. 2019). Genetic adaptation to climate in the western United States, or lack thereof, following a series of founder events from a single source area could be influencing the success of this biocontrol insect.

**FIGURE 1:**
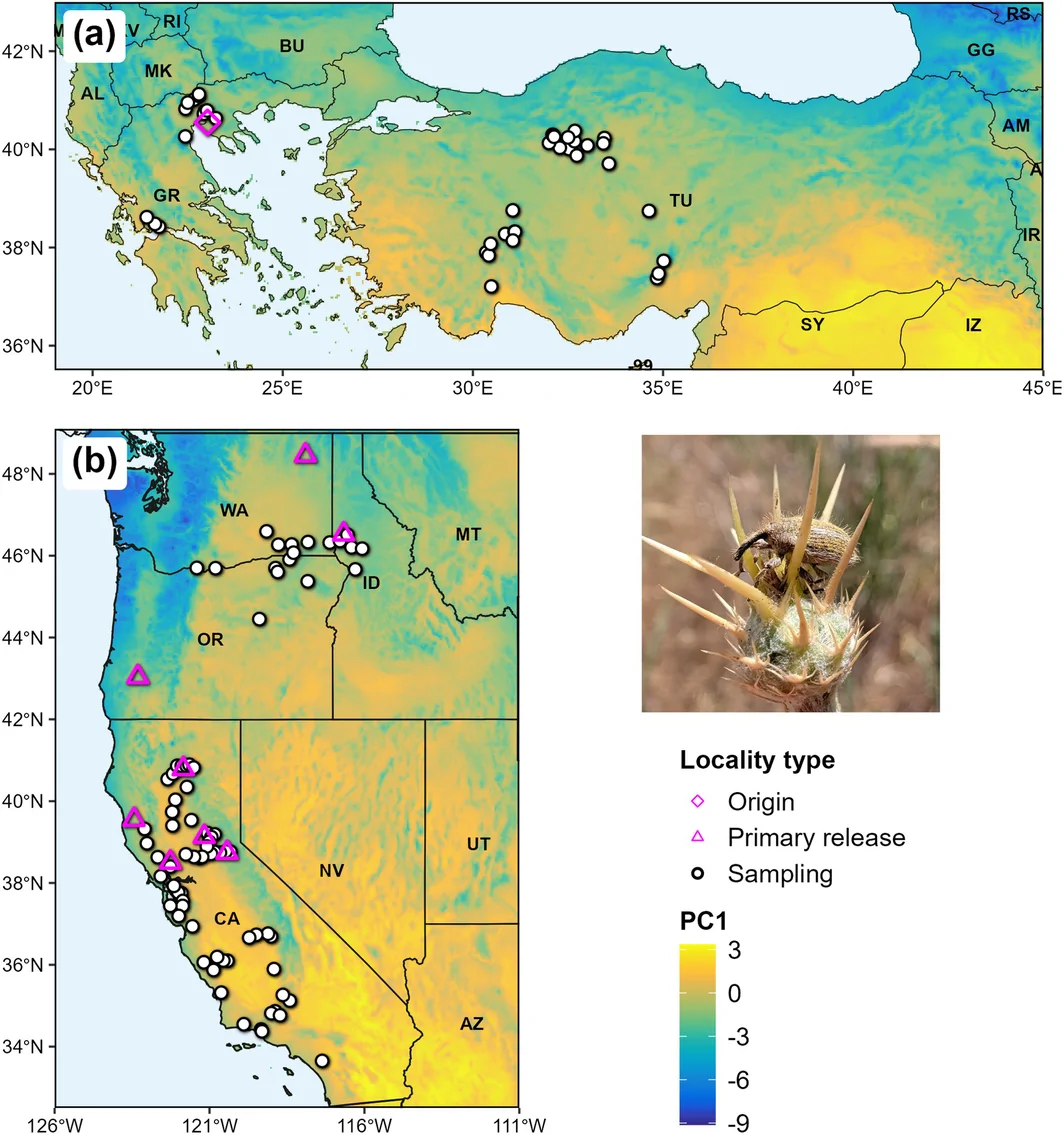
Sampling of *Eustenopus villosus* for this study in relation to climate and the insect's introduction history in the western United States. (a) Sampling sites in Türkiye and Greece, including the origin for all introduced populations in the United States (two sites near Thessaloniki, Greece). (b) Sampling sites in the western United States, including primary release sites. Climatic variation in each sampling region is represented by the first principal component (PC1) of a PC analysis of the five environmental predictors included in genotype‐environment association analyses. Inset photo depicts *E. villosus* on a developing flower bud of *Centaurea solstitialis* (photo by B.S. Barker).

We used a landscape genomic approach to infer local adaptation to climate in the native and introduced ranges of *E. villosus* and quantified the extent to which founder events impacted climate‐associated genetic variation in the introductions. First, we assessed population structure in *E. villosus* in sampled parts of its native range in Greece and Türkiye to inform genotype‐environment analyses (GEAs), accounting for background structure in allele frequencies to reduce false‐positive signals for local adaptation (De Villemereuil et al. 2014; Frichot et al. 2013; Whitlock and Lotterhos 2015). We then used six different GEA analyses to identify single nucleotide polymorphism (SNPs) associated with climate in the native range and explored the extent to which founder events in the western United States have affected levels of variation genome‐wide and at candidate loci associated with climate. We hypothesize that human‐mediated introduction and redistribution of insects, which resulted in both long‐distance “jump” dispersal from the source area in Greece and serial dispersal in the western United States, have reduced adaptive genomic variation associated with climate in the introductions. Specifically, we predicted that variation would be successively lost as insects were distributed across five sites between 1990 and 1991, and then subsequently redistributed to other areas, including in southern California and the Pacific Northwest.

Next, we tested whether climate‐associated candidate loci in *E. villosus* exhibit similar associations in the native and introduced ranges, or whether novel climate associations have evolved in the introduced range. Repeated association of the same loci with climate in the native and the introduced range may suggest that adaptive variation was introduced in the biocontrol agent and adapted in response to climate gradients in both regions under similar selective pressures. Support for this hypothesis would indicate that the initial founding population originating from Greece contained sufficient genetic variation associated with climate in the species and that adaptive evolution occurred despite serial introductions. Alternatively, if associations with climate are found for different SNPs, then this would suggest that local adaptation has occurred in different regions of the genome, either from new mutations or standing variation. Finally, no associations of genetic variation with climate in the introduced range would reveal a lack of realized adaptation in *E. villosus* biocontrol, and potential limitations on its current success. Thus, an understanding of the ability of weed biocontrol agents to evolve rapidly despite founder events has important implications for managing invasive plants across environmental gradients and under climate change (Harms et al. 2020; Szűcs et al. 2019; Wright and Bennett 2018).

## 2 Methods

### 2.1 Study System

Genus *Eustenopus* Petri, 1907 belongs to the tribe Lixini (Curculionidae: Lixinae) (Aslam 1963; Ter‐Minasian 1967), with four species distributed in the Palearctic region (Alonso‐Zarazaga et al. 2017). Most species of the Lixinae subfamily develop on plants belonging to the families Chenopodiaceae, Asteraceae, Brassicaceae, and Apiaceae (Ter‐Minasian 1967). The biological characteristics of *E. villosus* make it ideal for exploring genetic adaptation to climate. The beetle has a short generation time (1 year) and can attain high population densities (Fornasari and Sobhian 1993; Jetter et al. 2003), which are traits that may promote rapid adaptation of introduced species to new environments (Dlugosch, Anderson, et al. 2015; Szűcs et al. 2017, 2019). In California, *E. villosus* occurs at low abundances both in the very dry, low‐humidity regions of the Central Valley (Pitcairn et al. 2008) and in the cool, wet coastal areas of the San Francisco Bay Area (Swope and Satterthwaite 2012; Villegas 1997), with higher abundances documented in the Sierra Nevada foothills (Pitcairn et al. 2008). Warmer winter conditions have been linked to higher abundances in southeastern Washington (Woodley 2018). In its native range in Eurasia, *E. villosus* has a patchy distribution across climatically diverse areas in Bulgaria, Greece, Moldova, Ukraine, Türkiye, Syria, Iran, and the Caucasus (Figure 1; Clement et al. 1988; Fornasari and Sobhian 1993; Sobhian and Zwölfer 1985; Ter‐Minasian 1967). Coastal regions of the Mediterranean Basin have mild, wet winters and warm to hot, dry summers, whereas mountainous regions in the Caucasus (parts of Türkiye and Iran and the Russia‐South European Territory) have winter temperatures cold enough to support a period of snow each year and relatively moderate precipitation occurring mostly in the summer (Lionello et al. 2006). Thus *E. villosus* occupies a wide range of habitats in both of its ranges, with the potential for local adaptation in the introductions to enhance fitness and efficacy of biocontrol populations across a range of environments.

The distribution of *E. villosus* in the western United States has been shaped by both local and long‐distance dispersal. All populations originate from Doirani and Oreokastro, Greece, which we refer to as the source area because these two cities are geographically proximate (*ca*. 60 km apart; Figure 1) (Fornasari et al. 1991; Turner et al. 1994). Following inspection for parasites at a United States Department of Agriculture facility (Albany, California), a total of 1445 adult weevils were released at five sites [avg. 289 individuals per locality (range 200–400)] in northern California (Figure 1 and Data S1) between 1990 and 1991, where they successfully established (Villegas 1995). These populations served as sources for additional introductions in California, Oregon, Washington, and Idaho beginning 2–3 years after initial introductions (Pitcairn et al. 2008; Villegas 1995, 1997, 2000). In California, insects from the five initial release sites were released across 44 additional counties between 1994 and 1995, for a total of 105 releases in the state (Villegas 1997, 2000). In the Pacific Northwest, the earliest releases in Idaho occurred in 1995 at Nez Perce Indian Reservation (*N* = 528), in Oregon in the Myrtle Creek area of Douglas County in 1990 and 1991 (*N* = 204–314), and in Washington in 1996 near Kettle Falls in Stevens County (unknown number; Stevens County Noxious Weed Control Board n.d.) (Figure 1 and Data S1). These populations subsequently served as sources for additional releases in each state. Releases of *E. villosus* continue to the present day, with some agencies, such as Nez Perce Tribe's BioControl Center in Idaho, continuing to document release numbers and locations (Nez Perce Soil and Water Conservation District 2026). The dispersal capabilities of *E. villosus* have not been formally documented; however, field observations suggest it spreads more slowly than *Bangasternus orientalis*, another seed‐feeding biocontrol agent of *C. solstitialis* (Fornasari and Sobhian 1993; Swope and Satterthwaite 2012; Wilson et al. 2003).

### 2.2 Sampling and Genome‐Wide Sequencing

We sampled adult specimens of *E. villosus* from 40 sites in the native range (Greece and Türkiye) and 81 sites in the introduced range in the western United States (California, Oregon, Washington, and Idaho) between 2012 and 2015 (Figure 1, Table S1 and Figure S1). We attempted to maximize environmental differences between sites while minimizing geographic distance to increase the statistical power of GEAs while limiting false positive associations (Dauphin et al. 2023; Forester et al. 2018; Rellstab et al. 2015). Study sites spanned broad elevational (4–1705 m) and latitudinal (33.6°–46.6° N) gradients across both regions (Table 1), representing areas with hot and dry summers and milder winters (e.g., western Greece and central California) to areas with continental climates that experience freezing winters and snowfall (e.g., Anatolia and the eastern Pacific Northwest). Sampling other parts of the agent's native range was infeasible due to resource and access limitations. Specimens were stored in 95% ethanol. At least two individuals were collected from each site.

**TABLE 1:** Summary of sampled populations of *Eustenopus villosus*. The number of sites, total number of individuals (*N*_tot_) sequenced after quality filtering, average number of individuals sequenced per site after quality filtering (*N*_avg_), and average and range of values for elevation and climatic conditions according to five environmental predictors for the entire range, as well as regions within each range, are indicated.

	Native range			Introduced range				
	All	Greece	Türkiye	All	California	Oregon	Washington	Idaho
Sites	40	13	27	81	63	6	7	5
*N*_tot_	101	32	69	202	151	16	22	13
*N*_avg_	2.5	2.5	2.6	2.5	2.4	2.7	3.1	2.6
Elevation (m)	731	168	1002	519	520	550	363	696
	(69–1621)	(69–347)	(616–1621)	(4–1705)	(4–1705)	(254–903)	(166–816)	(474–1100)
Mean diurnal range (°C) (bio2)	11.9	11.3	12.2	14.4	14.7	13.9	12.5	13.6
	(9.8–13)	(10.3–12.2)	(9.8–13)	(11.2–19.1)	(11.2–19.1)	(12.7–16.5)	(11.5–13.2)	(12.9–14.3)
Maximum temperature of warmest month (°C) (bio5)	30.6	32.1	29.9	31.8	32.1	30.9	30.4	31.1
	(27.8–33.1)	(30.8–33.1)	(27.8–32.4)	(24.5–38.9)	(24.5–38.9)	(29.5–31.8)	(28.4–32.3)	(28.2–32.7)
Minimum temperature of coldest month (°C) (bio6)	−2.3	0.2	−3.5	0.3	1.4	−3.8	−2.7	−4.3
	(−5.7–3.3)	(−1.2–3.3)	(−5.7 – −1.1)	(−6.8–5)	(−5.2–5)	(−5.8 – −2.9)	(−4.3 – −1.9)	(−6.8 – −3.1)
Precipitation of driest quarter (mm) (bio17)	52	74.1	41.6	25	16.0	46.0	43.6	87.8
	(26–87)	(40–87)	(26–59)	(2–104)	(2–49)	(31–67)	(21–59)	(71–104)
Precipitation of coldest quarter (mm) (bio19)	169	156	175	320	371	109	160	157
	(100–332)	(100–332)	(113–274)	(69–801)	(124–801)	(82–142)	(69–352)	(116–209)

Genomic DNA was isolated from *E. villosus* samples using a modified phenol‐chloroform protocol (Chen et al. 2010). Most DNA isolations underwent an additional purification step with Serapure (Faircloth et al. 2014) to remove a contaminant(s) that inhibited restriction enzyme digests involved with ddRADseq library construction. Specimens with evidence of severe DNA degradation, which may have occurred due to exposure to warm temperatures during field collection, were excluded from sequencing. We used a modified version of Peterson et al.'s (2012) ddRADseq protocol to construct reduced‐representation libraries of genomic DNA for a total of 379 *E. villosus* specimens. Following fluorometric quantification (Qubit 2.0 high‐sensitivity or broad‐range assay, Invitrogen), 350–500 ng of each sample was digested with *MseI* and *EcoRI*. Unique individual P1 barcoded adapters (*N* = 83) were annealed to genomic DNA of each sample [P1 adapter top oligo: 5′AATGATACGGCGACCACCGA/GATCTACACTCTTTCCCTACACGACGCTCTTCCGATCT/xxxxxx/3′ (xxxxxx = barcode); P2 adapter as in Barker et al. (2017) except with no barcode]. Barcodes used to differentiate multiplexed individuals were six base pairs long and differed by at least two nucleotides. Barcoded DNA was pooled, purified using either AmpureXP beads (Beckman Coulter Inc.) or Serapure, and size selected to 410–590 bp using Pippin Prep automated DNA size selection system (Sage Science, Beverly, MA, USA).

We enriched adapter‐ligated fragments in size‐selected libraries using 12 PCR cycles, purified the resulting product using AmpureXP beads (Beckman Coulter Inc., Brea, CA, USA), and pooled samples into nine sequencing runs (36–80 samples per run). Pooled libraries were sequenced on the Illumina HiSeq 2500 or NextSeq 500/550 platforms (Illumina Inc., San Diego, CA, USA) to generate 100 or 150 bp paired‐end reads, respectively. Several samples (*N* = 141) were sequenced 2–5 times to increase the number of reads. Accessions and run information are available in NCBI under BioProject ID PRJNA275994.

### 2.3 Data Processing

Raw reads were trimmed to 100 bp for consistency of read length as required in STACKS by trimming the 5′ ends of reads > 100 bp with Trimmomatic v0.39 (Bolger et al. 2014). The trimmed reads were demultiplexed and cleaned in STACKS v2.60 (Catchen et al. 2013, 2011) using “process_radtags” with ‐c, ‐q, and ‐r flags allowing for a single mismatch in the restriction enzyme site. Read sets belonging to the same sample but sequenced on multiple lanes were then concatenated into a single file. Due to the low quality of the reverse reads in many samples, we retained only the forward sequencing reads.

The “denovo_map.pl” pipeline program was employed to merge stacks (i.e., sets of identical reads) into RAD loci within individuals, identify polymorphic sites, create a catalog of loci across individuals, determine the allelic state at each locus in each individual, and exclude reads with likely sequencing errors. We inferred loci using a minimum coverage depth of five (‐m 5) to create a stack, a maximum mismatch distance of two nucleotides between loci when processing a single individual (‐M 2), and a maximum of two stacks at a single locus (‐X 2). We selected these parameters after testing several different “M” and “n” (the number of mismatches permitted when matching stacks across individuals) values for evidence of errors in merging or splitting across loci (Figure S2). We then removed 76 samples with low coverage, defined as those with one or more standard deviations less than the mean number of loci recovered across all samples (< 23,028 loci). The dataset was thinned in VCFtools v0.1.16 (Danecek et al. 2011) to retain only one SNP per locus (‐‐thin 500) and biallelic sites with a maximum of 25% missing data and minor allele frequency greater than 0.01 (‐‐max‐missing 0.75, ‐‐max‐alleles 2, ‐‐maf 0.01). We also tested for heterozygote excess following Wigginton et al. (2005) (‐‐hardy) and removed sites with a significant excess of heterozygotes (*p* < 0.05). After filtering, our final SNP dataset included 7087 SNPs across 303 individuals, with a mean of 2.5 individuals per site (range: 1–5; Table S1).

### 2.4 Population Structure

We examined population structure in our dataset to identify divergent geographic regions and inform our GEA models (see “2.6 Genotype‐environment associations”). Prior to analyzing population structure, we conducted a preliminary scan for SNPs that may be under selection using BayeScan v2.1 (Foll and Gaggiotti 2008), as unique patterns of variation at these loci could obscure underlying population structure. We defined large geographic regions (native: Greece and Türkiye; introduced: western United States) as the populations used by BayeScan with 5000 iterations, 20 pilot runs each of length 5000, and burn‐in length of 50,000. No SNPs were found to have significantly different allele frequencies; therefore, we retained all SNPs in examining population structure.

To assess population structure, we performed a PCA using the “dudi.pca” function in ade4 v1.7‐23 (Dray et al. 2007) in R v4.4.1 (R Core Team 2024), retaining the first 50 PC axes. We further used ADMIXTURE v1.3.0 (Alexander et al. 2009) with *K* values from 1 to 15, with the optimal *K* value determined by selecting the value with the lowest cross‐validation error.

### 2.5 Environmental Data and Climatic Niche Comparisons

Bioclimatic variables from the WorldClim dataset (Fick and Hijmans 2017) (https://www.worldclim.org; accessed 31 December 2025) at 30s resolution were used to examine the climatic niche space occupied by native and invading populations, and for the GEAs. Presence records for *E. villosus* were derived from this study and from the Global Biodiversity Information Facility (GBIF, http://gbif.org, accessed on 13 May 2025) (GBIF.org 2025) using rgbif v3.8.1 (Chamberlain et al. 2017), the literature, museum records, personal collections, and release records for the western United States (Table S2). We generated shapefiles for the native and introduced ranges from subsets of Natural Earth Data (https://www.naturalearthdata.com; accessed 31 December 2025) to crop a raster of the bioclimatic variables. For the native range, we used the countries which had occurrence data for *E. villosus* . For the introduced range, we used the states with known populations of the insect (California, Oregon, Washington, Nevada, and Idaho).

We estimated correlations among the 19 variables in both regions together and generated a PCA of the 19 bioclim variables across both the native and introduced range with the “princomp” function in R. We examined the loadings of each variable on the PCA to inform our selection of variables that were highly loaded on major PC axes and thus captured major axes of environmental variation across the distribution. Among these, we chose minimally correlated (*r* < 0.8) variables that would be most likely to limit the survival of *E. villosus* for downstream analyses. The metric values were summarized to quantify environmental variation in each range independently.

Five climatic variables were ultimately retained for all further analyses, and each is expected to play an important role in the biology of *E. villosus* . Maximum temperature of warmest month (Bio5) and minimum temperature of coldest month (Bio6) reflect extremes in temperature that would limit the insect's distribution in very cold and hot areas. Adult *E. villosus* overwinter in soil litter and cracks in the soil surface and could therefore be impacted by freezing conditions even during diapause (Fornasari and Sobhian 1993). Precipitation of coldest quarter (Bio19) and precipitation of driest quarter (Bio17) may indirectly affect *E. villosus* through their impacts on *C. solstitialis* , which has reduced overwintering survival rates when exposed to prolonged freezing temperatures and ice accumulation https://www.zotero.org/google‐docs/?j36ktb (Roché et al. 1997; Gutierrez et al. 2017), which could reduce food for *E. villosus* . Likewise, prolonged and severe droughts can lead to high plant mortality (Enloe et al. 2004; Sheley and Larson 1994). Finally, mean diurnal range [mean of monthly (max temp − min temp)] (Bio2) captures the variation in temperatures experienced across *E. villosus* 's distribution. Sampling of *E. villosus* spanned a wide range of values for each of the five bioclimatic variables in both the native and introduced range (Table 1).

We used the R package ecospat v4.1.2 (Di Cola et al. 2017) to compare and classify any change in the climatic niche of *E. villosus* between the native and introduced ranges. First, we checked for spatial auto‐correlation between the bioclimatic variables with a Mantel test using the function “ecospat.mantel.correlogram”. Then we used the scripts from Broennimann et al. (2012) to prepare the occurrence and bioclimatic data, which includes thinning occurrences; we only retained occurrence points at least 200 m apart from each other based on the Mantel test. A PCA of the five bioclimatic variables calibrated on both ranges was generated with the “PCAenv” function in ecospat. The overlap of the climatic niche of the native and introduced ranges was calculated using Schoener's *D* (Schoener 1970) and we used the centroid, overlap, unfilling, and expansion (COUE) framework (Guisan et al. 2014) to classify changes in niche dynamics: changes in the centroid (a change in the mean position of climatic niche between two ranges), overlap (intersection between native and introduced niche), expansion (niche space of the introduced range that is not occupied in the native range), unfilling (niche space that is occupied in the native range but not the introduced range) of niches between ranges. Niche similarity and niche equivalency tests were performed with 1000 replicates each with the following alternative hypotheses: overlap.alternative = “lower”, expansion.alternative = “higher”, stability.alternative = “lower”, unfilling.alternative = “higher”.

### 2.6 Genotype‐Environment Associations

To determine which loci are involved in adaptation to climate in both the native and introduced range, we performed a series of complementary GEAs which test for associations of allele frequencies and environmental gradients. The total dataset was split into two: one containing only the introduced populations and one containing only the native populations. We applied the following six complementary approaches to identify genotype‐environment associations while reducing reliance on the assumptions and limitations of any single method: redundancy analysis (RDA), partial RDA (pRDA), Bayesian hierarchical model (Bayenv), latent factor mixed models (LFMM), and gradient forest, including gradient forest on the raw allele frequencies (GF) and on the standardized allele frequencies (GF‐X). These methods differ in their statistical frameworks, treatment of population structure, and ability to detect multivariate, linear, and nonlinear associations (see detailed comparisons in Table 2). Four of the methods (pRDA, Bayenv, LFMM, and GF‐X) accounted for population structure, which may confound GEA analyses by creating false, spurious correlations between genetic markers and environmental variables due to shared ancestry rather than local adaptation (De Villemereuil et al. 2014; Frichot et al. 2013; Whitlock and Lotterhos 2015). A detailed description of methods for the six GEA analyses is available in Methods S1.

**TABLE 2:** Comparison of genome–environment association methods used to identify climate‐associated genomic variation in *Eustenopus villosus*. The six approaches differ in their statistical frameworks, treatment of population structure, and ability to detect multivariate, linear, and nonlinear associations between genetic variation and environmental variables.

Feature	RDA	pRDA	Bayenv	LFMM	GF	GF‐X
General approach	Multivariate ordination	Partial multivariate ordination	Bayesian	Latent‐factor mixed model	Random forest	Random forest
Association type	Multivariate	Multivariate	SNP × environment	SNP × environment	SNP × environment	SNP × environment
Jointly analyzes environmental variables	Yes	Yes	Generally no	Generally no	Yes	Yes
Accounts for population structure	No	Yes	Yes	Yes	No	Yes
Method for accounting for population structure	—	Conditioning variables	Population covariance matrix	Latent factors	—	Correction/standardization based on population structure
Linear relationships	Yes	Yes	Yes	Yes	No	No
Nonlinear relationships	No	No	No	No	Yes	Yes
Interactions among environmental variables	Yes	Yes	Limited	Limited	Yes	Yes
Multivariate genetic response	Yes	Yes	No	No	Yes	Yes
Primary strength	Detects multivariate associations	Separates environmental effects from population structure	Models covariance among populations	Controls unobserved population structure	Captures nonlinear relationships	Captures nonlinear relationships while accounting for population structure
Important limitation	Sensitive to population structure and collinearity	Depends on appropriate conditioning variables	Computationally intensive; assumptions about covariance	Choice of latent‐factor number; univariate	Does not inherently account for population structure	More complex interpretation and implementation

Abbreviations: Bayenv, Bayesian environmental association analysis; GF, Gradient Forest; GF‐X, Gradient Forest accounting for population structure; LFMM, latent factor mixed models; pRDA, partial redundancy analysis; RDA, redundancy analysis.

### 2.7 Serial Founding Events and Genomic Diversity

We estimated and compared population genomic diversity in *E. villosus* across all sampling localities to test whether genomic diversity was successively lost as insects from the source area (Thessaloniki, Greece) were distributed across the western United States. First, we inferred the release history of sampled populations in the western United States from state weed biocontrol program managers and the literature (Data S1). Data for Oregon and Idaho were the most comprehensive, with details on sites (geographic coordinates), release dates, source populations, and the approximate number of insects released. Conversely, data for California consisted only of release summaries for entire counties (years and number of release sites) except for the five initial release sites. Records were completely absent for Washington except for a single site; therefore, we excluded samples from Washington from analyses of serial founding events.

Sampling sites were classified into one of six categories that describe their region of origin (Türkiye or Greece) or release history in the western United States (Primary, Secondary, Tertiary, or Quaternary; Figure S3). We assumed that each sampling site shared the same introduction history as the closest release record, which was identified by creating a sequence of 5‐km circular buffers ranging from 5‐ to 75‐km for each locality. Introduced populations were separated into 14 geographic groups, each of which shared a similar introduction history (Figure S3), to increase population sample sizes for population genomic analyses (average N per group = 11.2, range = 5–24). We attempted to account for uncertainty of release histories of *E. villosus* from several sampled sites by assigning individuals to broad introduction history categories for genetic diversity analyses (e.g., a mix of Primary, Secondary, and Tertiary).

For each classification scheme, we grouped all individuals in each category and calculated expected and observed heterozygosity (H_E_ and H_O_, respectively), inbreeding coefficient (*F*_IS_), and allelic richness (A_R_) with hierfstat v0.5‐11 (Goudet 2005) and site‐based nucleotide diversity (π) with vcftools v0.1.16 (Danecek et al. 2011). These values were calculated separately for three sets of loci: candidate loci identified in the native range, candidate loci identified in the introduced range, and non‐candidate loci across both ranges. We focused on candidate loci identified by the pRDA analysis because this method detected more loci than other GEA approaches while also accounting for population structure (see “3.4 Genotype‐environment associations”).

## 3 Results

### 3.1 Dataset Construction and Evaluation

In total, we obtained 2.1 billion Illumina reads across 379 individuals. Processing with STACKS recovered an average of 72,160 loci per individual (±49,132 loci [standard deviation]), and 76 samples that had fewer than 23,028 loci (one standard deviation less than the mean) were removed. After filtering for missing data and excess heterozygosity, a total of 7087 SNPs across 303 individuals were retained in our dataset and were used in downstream analyses.

### 3.2 Population Structure

The PCA identified distinct genetic clusters of *E. villosus* , with individuals from Türkiye forming one cluster and samples from Greece and the introduced range in the western United States forming a separate cluster (Figure 2a). The cross‐validation error estimated by ADMIXTURE was lowest at *K* = 2, after which it continued to increase to the highest *K*‐value tested (*K* = 15; Figure S4). At *K* = 2–3, individuals from Türkiye were differentiated from those in Greece and the western United States. At *K* = 3, most individuals in Greece and the western United States exhibited mixed ancestry, though the third genetic cluster occurred at highest frequency in California individuals (Figure 2b), particularly those from southern California populations (Figure S5).

**FIGURE 2:**
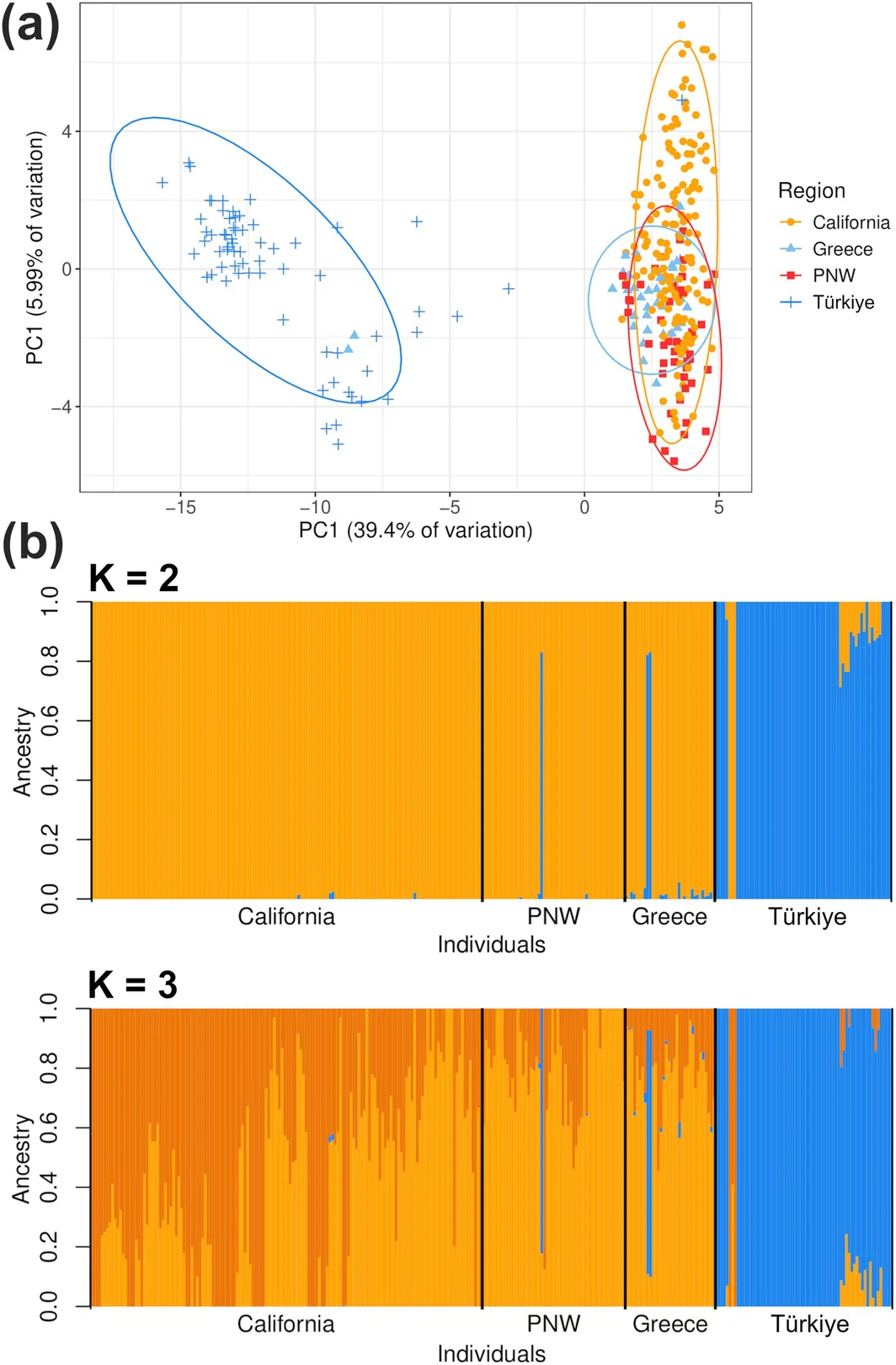
Population structure in *Eustenopus villosus* in its native (Greece and Türkiye) and introduced (western United States) range based on neutral single nucleotide polymorphisms. (a) Principal component analysis plot depicts clear clustering of individuals (dots) in introduced populations [warm colors = California and Pacific Northwest (PNW)] with native individuals from Greece (light blue), which are differentiated from those in Türkiye (dark blue). Ovals are 95% inertia ellipses. (b) Bar plots depict the ancestry proportions of 303 individuals according to ADMIXTURE analyses for *K* = 2 and *K* = 3, where *K* is the number of genetic clusters. PC1 = first principal component, PC2 = second principal component.

### 3.3 Climatic Niche Comparisons

A total of 927 records for *E. villosus* were thinned to 841 records from the native (*N* = 96) and introduced range in the western United States (*N* = 745; Figure S1). Across all bioclimatic variables, sampling sites included in genetic analyses in the introduced range spanned a wider range of climates than those in the native range (Table 1). Overall, native range sites were cooler and exhibited different seasonal precipitation patterns than introduced range sites (Table 1). Within the native range, sampling sites in Türkiye and Greece also differed in several temperature and precipitation variables (Table 1). Within the introduced range, sampling sites in the Pacific Northwest (i.e., Oregon, Washington, and Idaho) were generally cooler and wetter than those in California (Table 1). Analyses of change in the climatic niche of *E. villosus* between the native and introduced range indicated moderate niche overlap (Schoener's *D* = 0.618, *p* = 1.0) and high niche stability (stability factor = 0.874, *p* = 0.968) (Figure 3). Very little of the niche dynamics were classified as expansion (expansion factor = 0.125, *p* = 0.968) or unfilling (unfilling factor = 0.060, *p* = 0.937) and all niche differences were within analog space.

**FIGURE 3:**
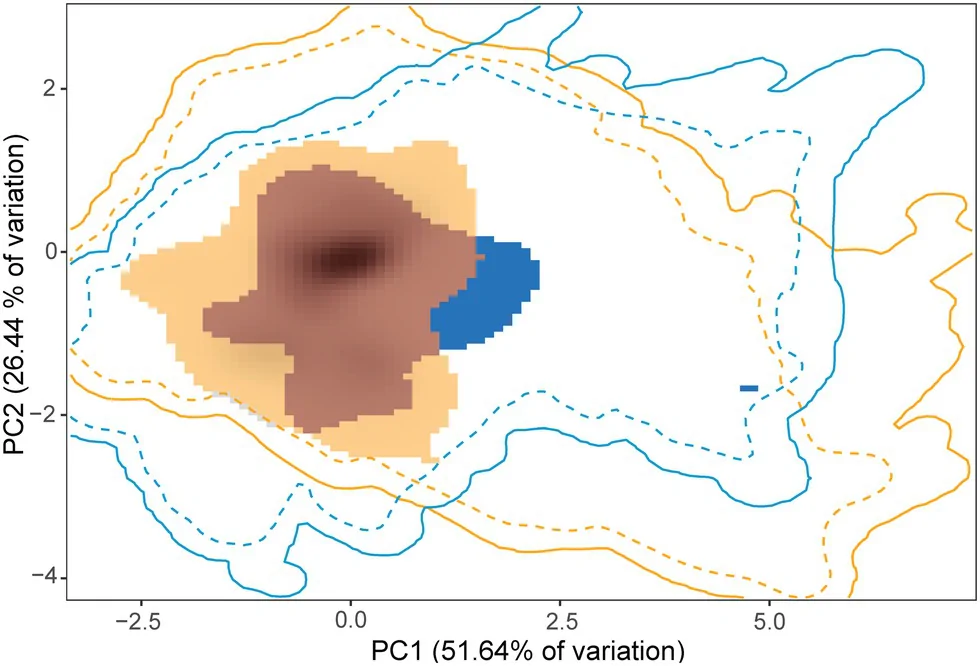
Comparison of the climatic niche for *Eustenopus villosus* in the native and introduced ranges based on a principal components analysis of five environmental predictors, which jointly explain 78.1% of the variance in the data. Solid lines on the periphery represent the extent of climate available in the native (blue) and introduced (orange) ranges, dashed lines represent 75% of the climatic space in each region, and fill areas represent the native, introduced, and shared (red) climatic niche space. Color darkness in filled areas corresponds to the density of observations (darker = more observations). PC1 = first principal component, PC2 = second principal component.

### 3.4 Genotype‐Environment Associations

The six complementary GEA methods (RDA, pRDA, Bayenv, LFMM, GF, and GF‐X) identified evidence of selection in both native and introduced populations of *E. villosus* , yielding a total of 664, 623, 307, 51, 472, and 86 candidate loci, respectively. Consistent with the different strengths and statistical approaches among methods, very few of these loci were shared (average = 2.02%, range = 0.0%–8.7%; Figure 4a). In the native range, RDA, pRDA, Bayenv, LFMM, GF, and GF‐X identified 335, 278, 134, 45, 330, and 84 candidate loci, respectively. Only one locus was shared among five of the six methods (Figure 4b). In introduced populations, the six methods identified 312, 291, 171, 6, 138, and 2 candidate loci, respectively. Although no locus was shared among all six methods, four loci were identified by four methods (LFMM, GF, RDA, and pRDA), with several additional loci shared among three methods. For populations in both ranges, RDA identified the largest number of candidate loci, whereas LFMM and GF‐X identified the fewest in the native and introduced ranges, respectively (Figure 5 and Table S3). Four methods (RDA, Bayenv, GF, and GF‐X) identified more candidate loci in native populations than introduced populations, with the largest disparity observed for LFMM (45 vs. 6 loci in native vs. introduced populations, respectively). This pattern should be interpreted cautiously particularly for RDA and GF, because these methods do not explicitly account for population structure in the native range and may therefore be more susceptible to false positives in this region.

**FIGURE 4:**
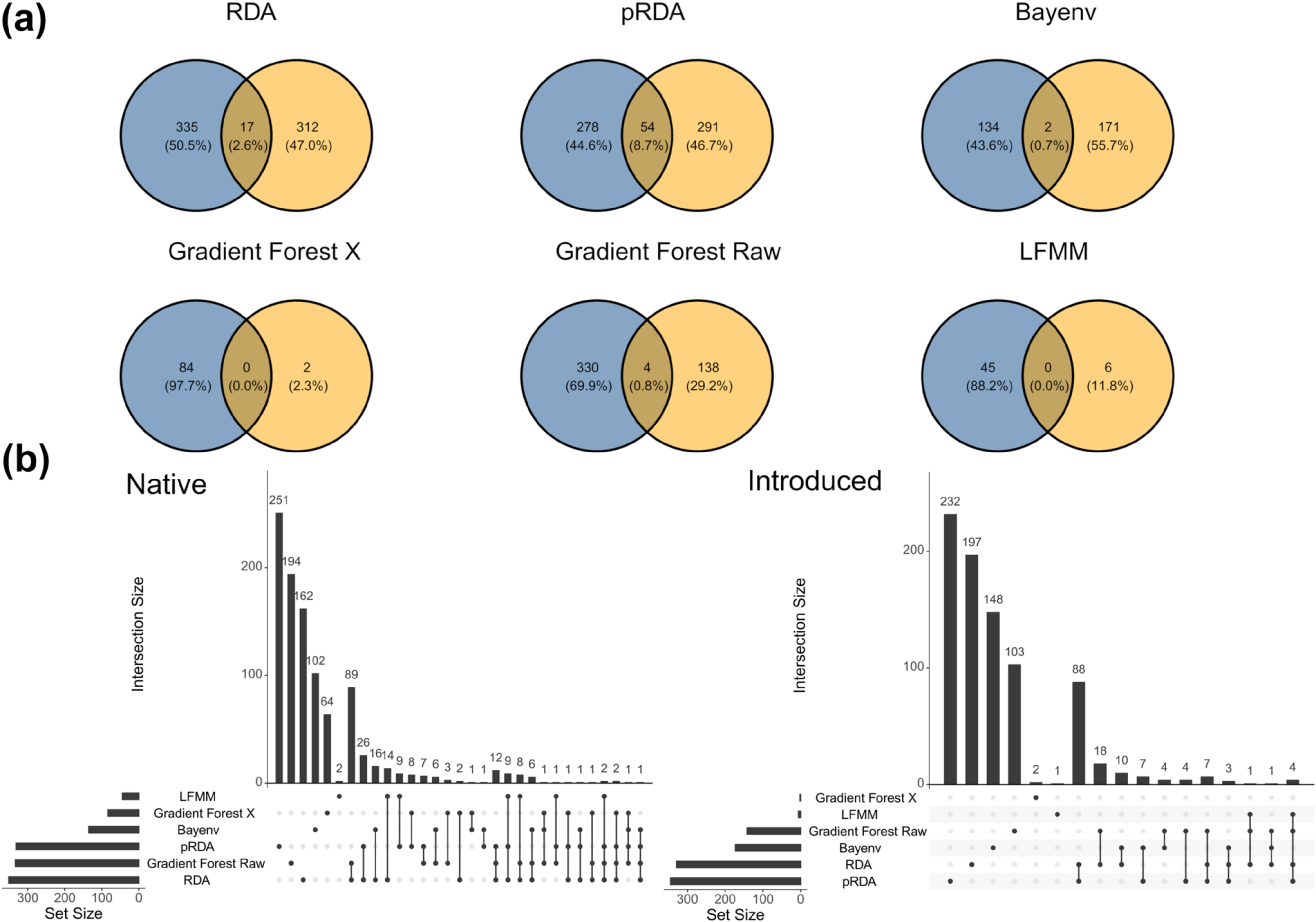
Candidate loci identified by six different genotype‐environment association (GEA) methods. (a) Venn diagrams showing the overlap of the candidate loci identified for the native (blue, left) and introduced (orange, right) ranges. (b) Upset plots show the intersections of the different GEA methods for the native and introduced range (left and right panels, respectively). Gradient Forest X, based on standardized allele frequencies from Bayenv; LFMM, latent factor mixed models; pRDA, partial redundancy analysis; RDA, redundancy analysis.

**FIGURE 5:**
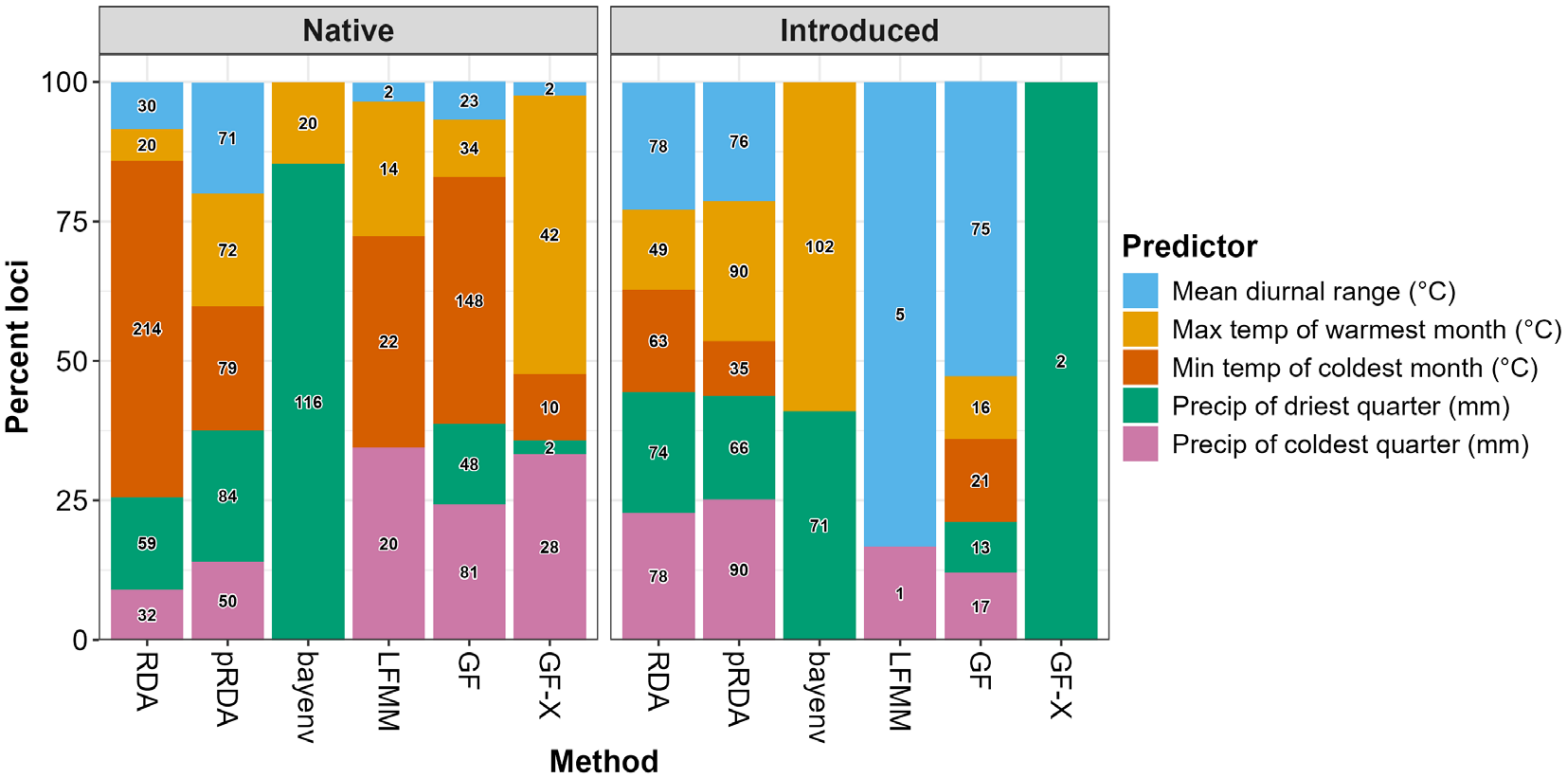
Number and proportion of loci associated with five environmental predictors in native and introduced populations of *Eustenopus villosus*, estimated using six genome–environment association methods: redundancy analysis (RDA), partial redundancy analysis (pRDA), Bayenv, latent factor mixed models (LFMM), Gradient Forest (GF), and Gradient Forest using standardized allele frequencies from Bayenv (GF‐X). Stacked bars show the proportional contribution of each predictor to the total loci identified by each method, while numbers within segments indicate the corresponding counts of candidate loci.

The number of loci associated with each environmental predictor differed between native and introduced populations (Figure 5). On average, more loci were associated with minimum temperature of the coldest month and precipitation of the driest quarter in native populations than in introduced populations (Table 3), whereas introduced populations exhibited more associations with mean diurnal range and maximum temperature of the warmest month. A similar number of loci were associated with precipitation of the coldest quarter in populations in both ranges. Associations also varied considerably among GEA methods, reflecting differences in the environmental relationships captured by each approach. For example, Bayenv identified numerous loci associated with maximum temperature of the warmest month and precipitation of the driest quarter for both ranges, whereas pRDA identified associations with a relatively even distribution of environmental predictors in introduced populations. GF ranked minimum temperature of the coldest month as the most important predictor in native populations and mean diurnal range as the most important predictor in introduced populations based on both accuracy and R^2^‐weighted importance (Figure S6). GF and GF‐X ranked precipitation of the coldest quarter as the second most important variable in both ranges based on accuracy importance.

**TABLE 3:** The average number (and range) of loci associated with five environmental predictors in native and introduced populations of *Eustenopus villosus* across six different genotype‐environment association methods.

Predictor	Native range	Introduced range
Mean diurnal range (bio2)	26 (2–71)	58 (5–78)
Maximum temperature of the warmest month (bio5)	34 (14–72)	64 (16–102)
Minimum temperature of the coldest month (bio6)	95 (10–214)	40 (21–63)
Precipitation of the driest quarter (bio17)	62 (2–116)	45 (2–74)
Precipitation of the coldest quarter (bio19)	42 (20–81)	46 (1–90)

### 3.5 Serial Founding Events and Genomic Diversity

Estimates of *A*_R_, π, and *H*_O_ were lower for non‐candidate loci than candidate loci identified in both the native and introduced range of *E. villosus* (Figure 6 and Table S4). In Türkiye, native‐range candidates had higher *A*_R_ (1.40), *H*_O_ (0.33), *H*_E_ (0.40), and *F*_IS_ (0.17) than the introduced‐range candidates (1.34, 0.29, 0.33, 0.14, respectively). These metrics were more similar between the two candidate sets in Greece, except that *F*_IS_ was 1.6× higher in native‐range candidates (0.16 vs. 0.10). Estimates of *F*_IS_ were higher in both sets of candidate loci than non‐candidate loci in native populations but were more variable across introduction‐history groups.

**FIGURE 6:**
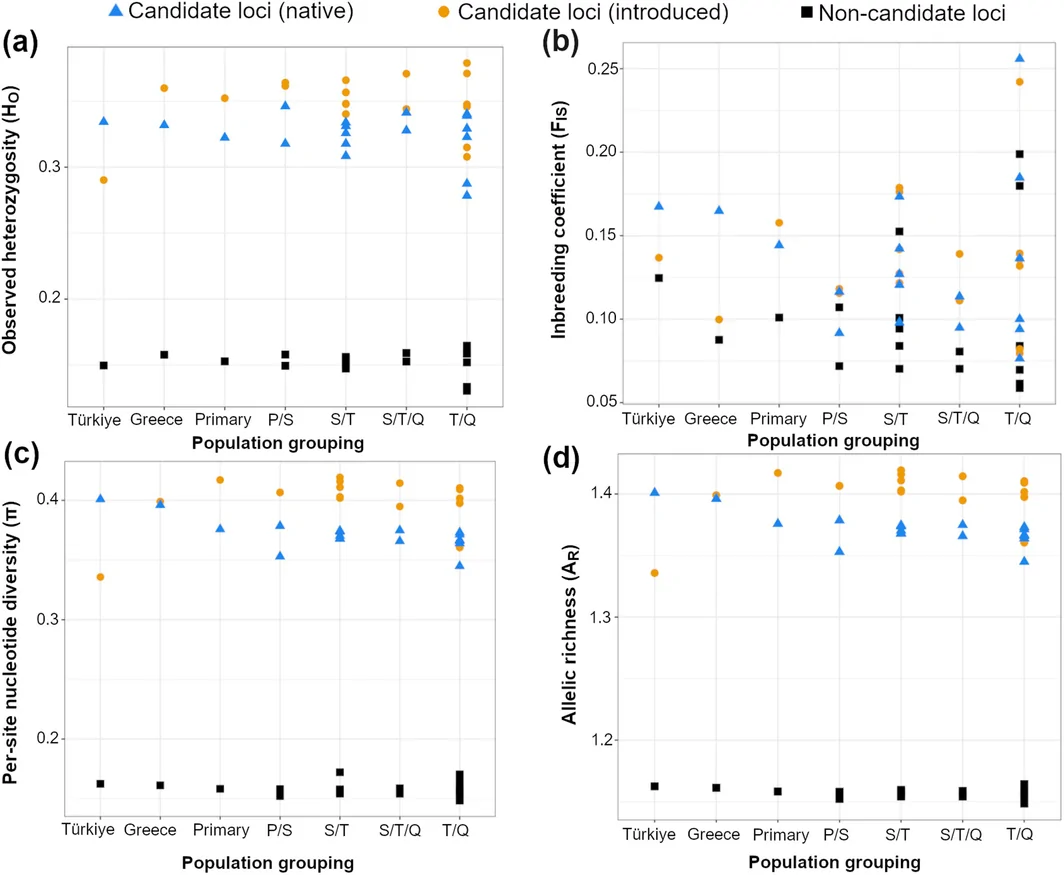
Climate‐associated and non‐associated genomic variation in *Eustenopus villosus*. (a) Observed heterozygosity (*H*_O_), (b) inbreeding coefficient (*F*_IS_), (c) per‐site nucleotide diversity (π), and (d) allelic richness (*A*_R_) of candidate loci in the native range (blue triangle), candidate loci in the introduced range (orange circle), and all other loci (black square), are shown for native population groups (Greece and Türkiye) and introduced groups classified by introduction history P, primary; P/S, primary/secondary; S/T, secondary/tertiary; S/T/Q, secondary/tertiary/quaternary; T/Q, tertiary/quaternary.

In general, there was little evidence for a decline in genetic diversity as *E. villosus* was redistributed across the western United States (Figure 6 and Table S4). Mean A_R_ and π at non‐candidate loci were slightly lower in the introduced range (1.37 and 0.16, respectively) than in Greece and Türkiye (both 1.40 and 0.16, respectively). However, estimates of *A*_R_, π, *H*_E_, and *H*_O_ were similar among introduction‐history groups across non‐candidate and candidate loci. *F*_IS_ in neutral and candidate loci was generally similar across introduced populations; however, estimates were highest in latest‐stage introductions (T/Q). Introduced‐range candidates exhibited higher *A*_R_, π, *H*_E_, and *H*_O_ in introduced populations than native‐range candidates.

## 4 Discussion

The role of standing genetic variation in promoting the establishment of species in new environments has been a persistent question in invasion biology (Baker and Stebbins 1965; Dlugosch, Anderson, et al. 2015; Sexton et al. 2009), with important implications for weed biocontrol effectiveness (Harms et al. 2020; Sentis et al. 2022; Szűcs et al. 2019). In biocontrol agents, adaptive genetic variation may facilitate responses to shifting environmental conditions (Roderick and Navajas 2003; Szűcs et al. 2019; Wright and Bennett 2018) as well as to the target plant species, which may themselves be evolving while invading (Barker et al. 2017; Colautti et al. 2010; Colautti and Lau 2015; Irimia et al. 2023). Here, we found evidence of climate‐associated genetic adaptation in *E. villosus* across its introduced range in the western United States despite serial founder events, wherein the agent was sourced from a single area in its native range and redistributed across the region. These findings suggest that the founding population retained sufficient climate‐related genetic variation for adaptive evolution to occur along climatic gradients during range expansion.

### 4.1 Population Structure

Analyses of population structure indicated differentiation between *E. villosus* populations in Greece and Türkiye, a finding that was also reported in a population genomics study of the host plant *C. solstitialis* (Barker et al. 2017). This spatially concordant differentiation is likely due to host specificity in *E. villosus* , geographic barriers to dispersal, and historical climate change. The peninsular region of Anatolia, also known as Asia Minor, is separated from the Balkan Peninsula (which includes Greece) by the Black Sea, the Marmara Sea and the Aegean Sea. Historical climate changes periodically fragmented and reconnected the ranges of species across the region, resulting in evolutionary divergence in numerous species of plants, fish, amphibians, and reptiles (Bilgin 2011; Hewitt 2011; Španiel and Rešetnik 2022).

Future work could assess the extent of co‐diversification of *C. solstitialis* and *E. villosus* in the native range, which includes much of Eastern Europe and the Caucasus, to evaluate its importance for biocontrol performance. Population genetic studies of biocontrol agents provide a framework for researchers to choose genetic groups for conducting tests of host‐specificity, efficacy, and differences in ecologically relevant traits (Gaskin 2024; Goolsby et al. 2006; Harms et al. 2020). Populations of *C. solstitialis* in the western United States are derived from different native source regions, with those in California arising from western Europe and exhibiting evidence for evolutionary increases in plant size and seed mass compared to native populations (Barker et al. 2017; Irimia et al. 2023). Conversely, plant populations in the Pacific Northwest are likely derived from multiple, independent introductions of divergent genotypes, though they are not well‐studied in terms of trait evolution (Barker et al. 2017). Although *E. villosus* co‐occurs with *C. solstitialis* across much of the western United States, its level of success on different host genotypes has not been evaluated. Biocontrol agents from the same native range as their host may be more effective due to their longer coevolutionary history, although in some cases, lineages with a shorter shared history can prove more effective (Goolsby et al. 2006; Hokkanen and Pimentel 1989; Manrique et al. 2014). Thus, more research is needed on the history of co‐diversification and its importance for biocontrol effectiveness.

### 4.2 Potential Adaptation to Climate

Our climatic niche analysis indicated that much of the climatic niche space for *E. villosus* in its native range was conserved in the introduced range in the western United States, with only a portion shifted from the niche space in the native range. This finding is consistent with evidence for climatic niche conservatism with *C. solstitialis* (Dlugosch, Cang, et al. 2015), as well as with research showing that introduced species of plants and insects often occupy similar climatic conditions in their introduced and native ranges (Hill et al. 2017; Liu et al. 2020; Riera et al. 2025). Niche similarity means that climate can be expected to exert similar selective pressures in both ranges, such that adaptive variation from the native range could enhance introduction success by establishing the same adaptive clines in the introduction (Colautti et al. 2010; Colautti and Lau 2015).

Although climatic niches were similar among the native and introduced ranges in this study, there were some differences that appeared to be reflected in the association of adaptive variation with climate variables. The distribution of *E. villosus* across hotter temperatures in the introduced range (particularly in California) compared to the native range may explain why a larger number of loci were associated with mean diurnal range and maximum temperature of the warmest month. Similarly, native populations spanned cooler environments in terms of the minimum temperature of the coldest month, with a larger number of loci associated with this predictor. Among GEA methods that accounted for population structure, the greater number of candidate loci in the native range may reflect the longer period over which *E. villosus* has evolved under local environmental conditions in the sampled areas. Increased sampling in both ranges would help determine whether this pattern is a product of history or potentially also a greater range of climatic adaptation in the native range. For example, sampling populations from climatically extreme parts of the native range, such as cold areas of the Caucasus and Russia or hot areas of the southern Mediterranean and Middle East, may reveal additional loci associated with climatic variables.

The GEA analyses revealed evidence for climatic adaptation at different loci in native and introduced ranges, which suggests that new associations have evolved during the agent's expansion in the western United States, even as the climatic niche was largely conserved. Additionally, this finding suggests that standing variation in *E. villosus* individuals originating from a single source area was sufficient for adapting to a range of climates. In general, our results are consistent with studies showing that rapid adaptation may occur in insects derived from a single source, provided that selection can act on beneficial alleles within the standing genetic variation of populations or new alleles that arise post‐invasion via mutation (Dlugosch, Anderson, et al. 2015; Dogantzis et al. 2024; Szűcs et al. 2019). These results are also consistent with a role for polygenic adaptation, in which natural selection acts on complex traits influenced by many loci of small effect, which GEA methods are well‐suited to detect (Dauphin et al. 2023; Forester et al. 2018; Rellstab et al. 2015).

The ability for biocontrol agents to adapt to climates in the introduced range despite originating from a small area (a narrow sampling of climate adaptations) has important implications for biocontrol programs. Some studies recommend releasing biocontrol agents in areas that climatically match their native origins, assuming pre‐adaptation to those conditions (Harms et al. 2020, 2021; Robertson et al. 2008). However, sampling an agent from multiple native range sites is often impractical or logistically infeasible. For example, the United States Department of Agriculture (USDA) Animal and Plant Health Inspection Service (APHIS) requires rigorous host‐specificity testing of agents from each site to ensure that the agent is specific to its target weed. Our findings suggest that *E. villosus* has adapted to climate during its range expansion, which may also explain why other agents, such as the feeding‐beetles *Galerucella calmariensis* L. and *G. pusilla* Duftschmidt (released to control *Lythrum salicaria* L.), have successfully established across wide climatic gradients despite originating from one or two areas in their native range (Hight et al. 1995; van Klinken et al. 2003). Additionally, they suggest that *E. villosus* might have the variation needed to successfully track *C. solstitialis* as the weed continues its range expansion in North America under current and future climates, including in the Intermountain West (Bradley et al. 2009; Pitcairn et al. 2006).

Differences in how the six GEA methods handle population structure, environmental dimensionality, and polygenic adaptation are likely to explain the low number of shared candidate loci among analyses (Forester et al. 2018; Lasky et al. 2023; Rellstab et al. 2015). GEA methods assume that adaptive alleles form frequency clines associated with the selective environment, but may produce false positives when allele frequency patterns do not reflect phenotypic clines associated with climatic gradients (Lotterhos 2023). Constrained ordinations (e.g., RDA) and mixed‐model/latent‐factor approaches (e.g., LFMM) generally provide a favorable balance of statistical power and false‐positive control (Forester et al. 2018; Frichot et al. 2013; Rellstab et al. 2015). Additionally, constrained ordinations perform well in systems characterized by weak population structure (Forester et al. 2018), as is the case for introduced *E. villosus* populations. Multivariate methods, especially RDA, have the ability to model multi‐dimensional relationships and detect weak polygenic signatures of selection (Forester et al. 2018; Rellstab et al. 2015). Conversely, LFMM may have lower detection rates for loci under weak or moderate selection (Forester et al. 2018), potentially contributing to its identification of the fewest number of candidate loci in this study. Thus, differences in candidate loci among methods should be interpreted as reflecting complementary and method‐dependent evidence for environmental associations rather than as conflicting results or definitive evidence of selection. Loci identified by multiple approaches provide stronger support for climatic adaptation, whereas method‐specific candidates should be considered putative and interpreted in light of the relative strengths of that method.

Several extensions of our work would enhance these inferences. The assembly of a reference genome for *E. villosus* would facilitate SNP identification and filtering, providing a more reliable variant dataset for GEA and enabling the identification of potential causal variants underlying climatic adaptation, such as genes under selection for increased cold or heat resistance (Leung et al. 2020; Pelosi et al. 2026), while genome resequencing and genome‐wide environmental association studies could further identify candidate genomic regions and help generate hypotheses about the molecular basis of adaptation (Lasky et al. 2023; Rellstab et al. 2015). Finally, assessing *E. villosus* performance under contrasting climatic conditions would help identify phenotypic traits underlying climatic adaptation, and linking variation in traits such as cold and heat tolerance with candidate genomic regions could provide stronger evidence that environmental associations reflect functional adaptation rather than environmental correlations (Lasky et al. 2023).

### 4.3 Serial Founder Events and Genetic Diversity

Founder effects are exacerbated in small populations (e.g., < 100 diploid individuals) owing to strong genetic drift, particularly when the population remains small for a number of generations (Nei et al. 1975). Thus, biocontrol insects reared in quarantine prior to release may be particularly at risk of losing genetic diversity (Fauvergue et al. 2012; Szűcs et al. 2019). Genetic diversity in *E. villosus* may have been maintained in the western United States due to the release of 1445 individuals following a brief quarantine period. The successful establishment of *E. villosus* at the five initial release sites in 1990 and 1991, followed by releases of 100 or more individuals at many additional sites (Data S1), may have further preserved genetic diversity by promoting rapid population growth during the early stages of introduction and thereby reducing founder effects (Nei et al. 1975). In general, our findings are consistent with theoretical predictions and empirical studies showing that larger founder numbers can prevent the loss of genetic diversity, potentially facilitating the establishment and range expansion of introduced species in novel environments (Dlugosch, Anderson, et al. 2015; Estoup et al. 2016; Uller and Leimu 2011).

Within the introduction, we sampled populations close to the agent's latitudinal (Washington and southern California) and longitudinal (eastern) range limits, with most of these belonging to later introduction history groups. We found no evidence for loss of variation towards these range margins or across serial founding events. The preservation of genetic diversity in these populations may suggest the potential for further evolution of local adaptation in the agent as its host continues to spread (Bradley et al. 2009; Pitcairn et al. 2006). We note that reduced effective population size of *C. solstitialis* at its range edge has not created a barrier to adaptation and further expansion of the plant (Braasch et al. 2019), which may expose *E. villosus* to additional novel selection from its host and climate.

Genetic variation was maintained across populations at both candidate and non‐candidate loci in *E. villosus* . Non‐candidate loci should be less influenced by selection and reflect neutral processes such as population bottlenecks and founding events. Candidate loci include the influence of selection, which can maintain favorable genetic variation during range expansion (Dlugosch, Anderson, et al. 2015; Estoup et al. 2016). We found markedly higher diversity in our candidate loci as compared to non‐candidate loci, which should be expected for loci with different allelic variants responsible for adaptation to different climates (the signature of selection identified by the GEA). This reflects the potential for spatially varying directional selection to maintain diversity at adaptive loci (Anderson et al. 2011). Candidate loci could also include those experiencing balancing selection in local environments, which would directly select for the maintenance of genetic variation within populations and also contribute to polygenic adaptation in heterogeneous environments (Dauphin et al. 2023; Forester et al. 2018). The elevated variation among our candidate loci reflects these effects and the maintenance of potential for further adaptation during range expansion.

A lack of detailed release records for most areas, particularly for Washington and much of California, hindered a full reconstruction of the introduction history for *E. villosus* . Therefore, our inferences of introduction histories at sampling sites could not account for potential undocumented releases or repeated introductions, which may help maintain or even increase genetic variation in introduced populations (Barker et al. 2017; Dlugosch, Anderson, et al. 2015; Uller and Leimu 2011). Additionally, understanding whether populations were products of releases that occurred after previous introductions had failed was hindered by an absence of data on establishment success at sampling sites, except for those located near the initial release sites. However, uncertainty about introduction histories is unlikely to change the interpretation of our results because genetic diversity was very similar across the introduction history groups used in our analyses, including at non‐candidate loci where variation is less likely to be maintained by selection and should reflect any founder effects. We note that without temporal sampling, we do not know for certain if genetic variation was simply maintained during founding events, or if gene flow after founding has also contributed to the maintenance of genetic variation.

## 5 Conclusions

Our work advances understanding of the extent and types of climate‐associated genetic variation in biocontrol agents derived from a limited portion of their native range. A preservation of standing genetic variation in *E. villosus* in the western United States likely facilitated its adaptation to climates during the agent's geographic expansion. This variation was likely maintained due to large release sizes, minimal quarantine duration, and successful establishment at the initial release sites. The capacity for adaptation despite limited native‐range sourcing is encouraging, given the logistical challenges of matching climates by introducing agents from multiple source regions. More broadly, identifying the evolutionary factors underlying geographic variability in biocontrol success could improve strategies for sourcing and releasing new agents, while also informing expectations for how existing agents may respond to novel and changing conditions.

## Funding

This work was supported by National Institute of Health Award #K12GM000708 through the Center for Insect Science at the University of Arizona to B.S.B., U.S. Department of Defense Strategic Environmental Research and Development Program (U.S. Army Corps of Engineers, contract #RC23‐3611) and U.S. Department of Agriculture (USDA) National Institute of Food and Agriculture (NIFA) awards (#2022‐68013‐37138) to B.S.B., a USDA NIFA Postdoctoral Fellowship (#2024‐67012‐43394) to J.A.P., and U.S. National Science Foundation (#1750280) and USDA NIFA (#2023‐67013‐40169) awards to K.M.D.

## Conflicts of Interest

The authors declare no conflicts of interest.

## Supporting information


**Data S1:** Release records for *Eustenopus villosus* acquired and analyzed for this study. Geographic information includes state, county, locality, latitude and longitude. Coordinates for records with only county‐level information (CountyLevel = 1) are county centroids. Release information may include the number of individuals released (NumRel), release date (RelDate), release year (RelYear), source population (SourcePop). For California, information may also include whether the population was part of redistribution efforts in California between 1990 and 1999 (Distrib90‐99), whether insects had established by 1995 (EstBy95), and the relative density of the population in 1999 (RelDens99). Rank indicates the release status assigned to the record for use in grouping populations in population genomic analyses (*P* = primary, S = secondary, T = tertiary, and Q = quaternary).

**Methods S1:** Description of the six genotype‐environment analysis methods used to identify SNPs associated with climate in *Eustenopus villosus* in the native and introduced range.
**Table S1:** Sampling information for *Eustenopus villosus* specimens included in population genomic analyses (*N* = 303) after quality filtering of genomic data. The range of origin (Range, native or introduced), country or state code (Region), province or county (ProvinceCounty), nearest city (NearestCity), site code (SiteCode), latitude, and longitude, elevation in meters above sea level (Elev), year and date of collection (Year, Date), and number of individuals (N) are indicated for each sampling site.
**Table S2:** Presence records for *Eustenopus villosus* from its native range derived from the literature, museum records, personal communications, and this study. The country, province, locality, latitude, longitude, date of collection (if known), and reference are indicated for each record. Geographic coordinates were estimated from city names for records lacking this information (Estimated = 1).
**Table S3:** Summary of loci associated with environmental predictors in native and introduced populations of *Eustenopus villosus*. The number of loci identified for each predictor as well as the total and average number identified across all five predictors is indicated for the redundancy analysis (RDA), partial redundancy analysis (pRDA), Bayenv, latent factor mixed models (LFMM), Gradient Forest (GF), and GF using standardized allele frequencies from Bayenv (GF‐X). Environmental predictors include: bio2 = mean diurnal temperature range (mean of monthly max temperature − min temperature), bio5 = maximum temperature of the warmest month, bio6 = minimum temperature of the coldest month, bio17 = precipitation of the driest quarter, and bio19 = precipitation of the coldest quarter. Note that the total number of candidate SNPs may not be equal to the sum of the SNPs associated with the environmental predictors because SNPs may be strongly associated with more than one predictor (e.g., in the RDA, a SNP can be classified as a candidate on more than one axis).
**Table S4:** Population genomic variation in *Eustenopus villosus*. Observed heterozygosity (H_O_), expected heterozygosity (H_E_), inbreeding coefficient (F_IS_), per‐site nucleotide diversity (π), and allelic richness (A_R_) are shown for (a) non‐candidate loci, (b) candidate loci in the native range, and (c) candidate loci in the introduced range. Groupings include the native geographic regions (Greece and Türkiye) and serial founding events in the introduced range. Founding classification: P = primary, P/S = primary/secondary, S/T = secondary/tertiary, S/T/Q = secondary/tertiary/quaternary, T/Q = tertiary/quaternary.
**Figure S1:** Geographic origins of records for *Eustenopus villosus* used for this study. The origin for all introduced populations in the western United States (two sites near Thessaloniki, Greece), primary release sites, sampling sites for genetic material, and sites used to classify the climatic niche for the native and introduced ranges are indicated.
**Figure S2:** Boxplots of the inbreeding coefficient (*F*_IS_) based on RAD loci in *Eustenopus villosus* individuals at different ‐*M* and ‐*n* values in STACKS, where ‐*M* is the number of nucleotide differences allowed between loci when processing a single individual and ‐*n* is the number of nucleotide differences allowed between loci when creating the catalog of loci. Boxplots show median (horizontal line), interquartile range (box), and minimum and maximum values (whiskers). Positive values for *F*_IS_ may indicate excess homozygosity due to failure to cluster loci under restrictive mismatch values, whereas negative values for *F*_IS_ may indicate excess heterozygosity due to clustering of paralogous loci.
**Figure S3:** Estimated introduction history and groupings of the sampled *Eustenopus villosus* populations in the western United States. Groups were classified by introduction history (P = primary, P/S = primary/secondary, S/T = secondary/tertiary, S/T/Q = secondary/tertiary/quaternary, T/Q = tertiary/quaternary). Populations in Washington were excluded from population genomic analyses owing to an absence of release records.
**Figure S4:** Plot of cross‐validation errors at different values of *K* (number of populations) according to ADMIXTURE analyses of single nucleotide polymorphism data for *Eustenopus villosus*.
**Figure S5:** Map with pie charts depicting the geographic distribution of three genetic clusters in introduced populations of *Eustenopus villosus* in the western United States. Pie slices indicate the proportion of each cluster. Pie sizes are proportional to the number of samples in the population.
**Figure S6:** Results of the gradient forest analysis on raw allele frequencies (GF) and standardized allele frequencies (GF‐X) for native (a, b) and introduced (c, d) populations of *Eustenopus villosus*. The accuracy importance and R^2^‐weighted importance of environmental predictors that explain allele frequencies are indicated for each analysis. Predictors include five bioclimatic variables: bio2 = mean diurnal temperature range (mean of monthly max temperature − min temperature), bio5 = maximum temperature of the warmest month, bio6 = minimum temperature of the coldest month, bio17 = precipitation of the driest quarter, and bio19 = precipitation of the coldest quarter.

## Data Availability

The ddRADseq data are deposited to the NCBI sequence read archive (BioProject #PRJNA275994 at https://www.ncbi.nlm.nih.gov/bioproject/).
