# Supplementary material for: Climate‐Associated Genetic Variation in Native and Introduced Populations of Eustenopus villosus, a Biological Control Agent for Yellow Starthistle
Source: Evol Appl. 2026 Nov 5;19(11):e70341. doi: 10.1111/eva.70341 (PMC13736000; doi:10.1111/eva.70341)
Supplement: Supplementary file 1 [file EVA-19-e70341-s001.docx]

**Methods S1.** Description of the six genotype-environment analysis methods used to identify SNPs associated with climate in *Eustenopus villosus* in the native and introduced range.

Redundancy analysis (RDA) and partial RDA (pRDA): The RDA and pRDA are multivariate genotype-environment analysis (GEA) methods that identify covarying allele frequencies associated with environmental factors that may result from polygenic responses occurring in complex environments [(Forester et al., 2018; Frichot et al., 2013; Legendre & Legendre, 2012)](https://www.zotero.org/google-docs/?bSlvdY). The pRDA used the first two principal component (PC) axes on the genetic dataset to condition and therefore correct for population structure. We used the ‘dudi.pca’ function in the R package ade4 to generate a principal component analysis (PCA) on each dataset independently, and the first two PC axes were used to condition the RDA within the ‘rda’ function in the R package vegan v2.6-10 [(Oksanen et al., 2025)](https://www.zotero.org/google-docs/?5W9b8n). The single nucleotide polymorphisms (SNPs) with the top 5% of loadings were classified as outliers with significant associations with the environmental variables. Given that each of the RDA axes explain unequal proportions of variance, we weighed the first three axes based on their inertia values (eigenvalues) such that the greater the inertia the greater the number of SNPs retained from that axis. The association of each candidate locus to environmental predictors was determined by examining the correlation of each candidate to each environmental predictor and taking the greatest correlation value.

Bayenv2: We used a Bayesian hierarchical model in Bayenv2, which calculates the posterior distribution (null model) from the empirical allele frequencies and then tests a regression between population allele frequencies and environmental factors against the null model [(Coop et al., 2010; Günther & Coop, 2013)](https://www.zotero.org/google-docs/?DHC9o9). First, we converted the introduced and native variant call format files to Bayenv format with PGDSpider v3 [(Lischer & Excoffier, 2012)](https://www.zotero.org/google-docs/?hPebcY), which removes any monomorphic sites, leaving 6,247 and 6,615 loci in the introduced and native ranges, respectively. Four independent runs of 100,000 iterations each were used to estimate the covariance matrix and the final matrix iterations from each run were exported and inspected in R. After verifying the similarity among the matrices, a single mean covariance matrix was generated that was used for downstream analyses. For each range, we standardized the environmental variables by subtracting the mean value and then dividing by the standard deviation. For each polymorphic SNP, we used Bayenv2 to calculate Bayes factors for the allele frequencies and the five standardized environmental predictors. Given that population history and outliers may bias the results of the model [(Günther & Coop, 2013)](https://www.zotero.org/google-docs/?wu6fmz), we also calculated the non-parametric metrics Spearman’s ⍴ and Pearson’s correlation coefficient for each SNP and environmental variable, which are based on standardized allele frequencies that adjusts for structure among populations [(Günther & Coop, 2013)](https://www.zotero.org/google-docs/?61r9N8). Loci with the top 5% of Bayes factors and top 7% of ⍴ were considered candidate loci.

Latent factor mixed models (LFMM): we applied the LFMM and univariate tests approaches to account for the nonlinear genomic responses to environmental predictors [(Ellis et al., 2012; Fitzpatrick et al., 2021; Fitzpatrick & Keller, 2015)](https://www.zotero.org/google-docs/?ifQq0w), while testing for a set of environmental predictors in a single model. The ‘lfmm_ridge’ and ‘lfmm_test’ functions in lfmm v1.1 [(Caye et al., 2019; Frichot et al., 2013; Jumentier et al., 2022)](https://www.zotero.org/google-docs/?j5N0Tv) were used to perform these analyses with the number of populations (K) set to one for the introduced range and two for the native range based on our PCA and ADMIXTURE results. The resulting P-values were adjusted using the genomic inflation factor and then corrected for multiple comparisons with a false discovery rate threshold (q-value < 0.1). Loci with a q-value < 0.1 were considered putative adaptive candidates.

Gradient forest (GF) and GF-X: Finally, we used GF, a machine learning GEA method that extends the Random Forest algorithm to detect allele frequency changes associated with environmental gradients [(Ellis et al., 2012; Fitzpatrick et al., 2021; Fitzpatrick & Keller, 2015)](https://www.zotero.org/google-docs/?WofgXB). Unlike the pRDA and LFMM, the GF method relies on population allele frequencies as the input for the genetic data. Raw population allele frequencies were generated with adegenet v2.1.11 [(Jombart, 2008; Jombart & Ahmed, 2011)](https://www.zotero.org/google-docs/?W8n3fd) and standardized allele frequencies were generated from the output of Bayenv2 [(Coop et al., 2010; Günther & Coop, 2013)](https://www.zotero.org/google-docs/?JMSYeN). We then used the gradientForest v0.1-37 R package [(Ellis et al., 2012)](https://www.zotero.org/google-docs/?A24JDu) to run a GF on the raw allele frequencies (GF) and on the standardized allele frequencies (GF-X). Of loci that had a positive R^2^ value, we took the top 5% and categorized them as candidate loci. The importance of each predictor was evaluated using the accuracy importance metric, which measures the decrease in model accuracy when a specific predictor is randomly permuted, as well as the R^2^-weighted importance metric, which weights the importance of each predictor by the proportion of variance explained.

**References**

[Caye, K., Jumentier, B., Lepeule, J., & François, O. (2019). LFMM 2: Fast and accurate inference of gene-environment associations in genome-wide studies. *Molecular Biology and Evolution*, *36*(4), 852–860. https://doi.org/10.1093/molbev/msz008](https://www.zotero.org/google-docs/?SozjVF)

[Coop, G., Witonsky, D., Di Rienzo, A., & Pritchard, J. K. (2010). Using environmental correlations to identify loci underlying local adaptation. *Genetics*, *185*(4), 1411–1423. https://doi.org/10.1534/genetics.110.114819](https://www.zotero.org/google-docs/?SozjVF)

[Ellis, N., Smith, S. J., & Pitcher, C. R. (2012). Gradient forests: Calculating importance gradients on physical predictors. *Ecology*, *93*(1), 156–168. https://doi.org/10.1890/11-0252.1](https://www.zotero.org/google-docs/?SozjVF)

[Fitzpatrick, M. C., Chhatre, V. E., Soolanayakanahally, R. Y., & Keller, S. R. (2021). Experimental support for genomic prediction of climate maladaptation using the machine learning approach Gradient Forests. *Molecular Ecology Resources*, *21*(8), 2749–2765. https://doi.org/10.1111/1755-0998.13374](https://www.zotero.org/google-docs/?SozjVF)

[Fitzpatrick, M. C., & Keller, S. R. (2015). Ecological genomics meets community‐level modelling of biodiversity: Mapping the genomic landscape of current and future environmental adaptation. *Ecology Letters*, *18*(1), 1–16. https://doi.org/10.1111/ele.12376](https://www.zotero.org/google-docs/?SozjVF)

[Forester, B. R., Lasky, J. R., Wagner, H. H., & Urban, D. L. (2018). Comparing methods for detecting multilocus adaptation with multivariate genotype–environment associations. *Molecular Ecology*, *27*(9), 2215–2233. https://doi.org/10.1111/mec.14584](https://www.zotero.org/google-docs/?SozjVF)

[Frichot, E., Schoville, S. D., Bouchard, G., & François, O. (2013). Testing for associations between loci and environmental gradients using latent factor mixed models. *Molecular Biology and Evolution*, *30*(7), 1687–1699. https://doi.org/10.1093/molbev/mst063](https://www.zotero.org/google-docs/?SozjVF)

[Günther, T., & Coop, G. (2013). Robust identification of local adaptation from allele frequencies. *Genetics*, *195*(1), 205–220. https://doi.org/10.1534/genetics.113.152462](https://www.zotero.org/google-docs/?SozjVF)

[Jombart, T. (2008). *adegenet*: A R package for the multivariate analysis of genetic markers. *Bioinformatics*, *24*(11), 1403–1405. https://doi.org/10.1093/bioinformatics/btn129](https://www.zotero.org/google-docs/?SozjVF)

[Jombart, T., & Ahmed, I. (2011). *adegenet 1.3-1*: New tools for the analysis of genome-wide SNP data. *Bioinformatics*, *27*(21), 3070–3071. https://doi.org/10.1093/bioinformatics/btr521](https://www.zotero.org/google-docs/?SozjVF)

[Jumentier, B., Caye, K., Heude, B., Lepeule, J., & François, O. (2022). Sparse latent factor regression models for genome-wide and epigenome-wide association studies. *Statistical Applications in Genetics and Molecular Biology*, *21*(1), 20210035. https://doi.org/10.1515/sagmb-2021-0035](https://www.zotero.org/google-docs/?SozjVF)

[Legendre, P., & Legendre, L. F. (2012). *Numerical Ecology* (Vol. 24). Oxford, UK: Elsevier.](https://www.zotero.org/google-docs/?SozjVF)

[Lischer, H. E. L., & Excoffier, L. (2012). PGDSpider: An automated data conversion tool for connecting population genetics and genomics programs. *Bioinformatics*, *28*(2), 298–299. https://doi.org/10.1093/bioinformatics/btr642](https://www.zotero.org/google-docs/?SozjVF)

[Oksanen, J., Simpson, G. L., Blanchet, F. G., Kindt, R., Legendre, P., Minchin, P. R., O’Hara, R. B., Solymos, P., Stevens, M. H. H., Szoecs, E., Wagner, H., Barbour, M., Bedward, M., Bolker, B., Borcard, D., Carvalho, G., Chirico, M., De Caceres, M., Durand, S., … Borman, T. (2025). *vegan: Community Ecology Package*. 2.6-10. https://doi.org/10.32614/CRAN.package.vegan](https://www.zotero.org/google-docs/?SozjVF)

**Table S1.** Sampling information for *Eustenopus villosus* specimens included in population genomic analyses (N = 303) after quality filtering of genomic data*.* The range of origin (Range, native or introduced), country or state code (Region), province or county (ProvinceCounty), nearest city (NearestCity), site code (SiteCode), latitude, and longitude, elevation in meters above sea level (Elev), year and date of collection (Year, Date), and number of individuals (N) are indicated for each sampling site.

| **Range** | **Region** | **ProvinceCounty** | **NearestCity** | **SiteCode** | **Latitude** | **Longitude** | **Elev** | **Year** | **Date** | **N** |
| --- | --- | --- | --- | --- | --- | --- | --- | --- | --- | --- |
| Native | Greece | Aetolia-Acarnania | Nafpaktos | Aet-01 | 38.423 | 21.748 | 347 | 2014 | 6/30/2014 | 3 |
| Native | Greece | Aetolia-Acarnania | Thermo | Aet-02 | 38.481 | 21.639 | 278 | 2014 | 6/30/2014 | 4 |
| Native | Greece | Aetolia-Acarnania | Agrinio | Aet-03 | 38.618 | 21.423 | 149 | 2014 | 6/30/2014 | 1 |
| Native | Greece | Kilkis | Vafiochori | Kil-01 | 41.015 | 22.67 | 69 | 2014 | 7/3/2014 | 3 |
| Native | Greece | Kilkis | Cherso | Kil-02 | 41.089 | 22.798 | 97 | 2014 | 7/3/2014 | 3 |
| Native | Greece | Kilkis | Dorsato | Kil-03 | 41.123 | 22.795 | 122 | 2014 | 7/3/2014 | 3 |
| Native | Greece | Pella | Pella | Pel-01 | 40.827 | 22.444 | 155 | 2014 | 7/3/2014 | 3 |
| Native | Greece | Pella | Stathis | Pel-02 | 40.952 | 22.502 | 130 | 2014 | 7/3/2014 | 3 |
| Native | Greece | Pierra | Pierra | Pie-01 | 40.262 | 22.436 | 92 | 2014 | 7/5/2014 | 3 |
| Native | Greece | Thessaloniki | Drimos | The-01 | 40.772 | 22.949 | 250 | 2014 | 7/3/2014 | 1 |
| Native | Greece | Thessaloniki | Oreokastro | The-02 | 40.726 | 22.928 | 249 | 2014 | 7/3/2014 | 3 |
| Native | Greece | Thessaloniki | Lagkadikia | The-03 | 40.633 | 23.238 | 113 | 2014 | 7/4/2014 | 1 |
| Native | Greece | Thessaloniki | Assiros | The-04 | 40.792 | 23.007 | 137 | 2014 | 7/4/2014 | 1 |
| Native | Türkiye | Adana | Pozantı | Ada-01 | 37.379 | 34.843 | 1124 | 2014 | 7/15/2014 | 2 |
| Native | Türkiye | Adana | Pozantı | Ada-03 | 37.471 | 34.884 | 1040 | 2014 | 7/17/2014 | 2 |
| Native | Türkiye | Afyon | Bolvadin | Afy-01 | 38.757 | 31.047 | 1040 | 2014 | 7/12/2014 | 1 |
| Native | Türkiye | Ankara | Akyurt | Ank-01 | 40.079 | 33.012 | 1000 | 2014 | 7/10/2014 | 3 |
| Native | Türkiye | Ankara | Dağdemir | Ank-04 | 40.23 | 33.458 | 846 | 2014 | 7/10/2014 | 3 |
| Native | Türkiye | Ankara | Kalecik | Ank-05 | 40.121 | 33.429 | 710 | 2014 | 7/10/2014 | 4 |
| Native | Türkiye | Ankara | Keskin | Ank-06 | 39.708 | 33.576 | 1094 | 2014 | 7/10/2014 | 3 |
| Native | Türkiye | Ankara | Yenikent | Ank-08 | 40.008 | 32.524 | 813 | 2014 | 7/11/2014 | 3 |
| Native | Türkiye | Ankara | Ayaş | Ank-10 | 40.032 | 32.285 | 757 | 2014 | 7/11/2014 | 4 |
| Native | Türkiye | Ankara | Beypazarı | Ank-11 | 40.129 | 32.001 | 616 | 2014 | 7/11/2014 | 4 |
| Native | Türkiye | Ankara | Doğançalı | Ank-12 | 40.296 | 32.093 | 1078 | 2014 | 7/11/2014 | 1 |
| Native | Türkiye | Ankara | Doğançalı | Ank-13 | 40.288 | 32.116 | 1038 | 2014 | 7/11/2014 | 1 |
| Native | Türkiye | Ankara | Uruş | Ank-14 | 40.256 | 32.133 | 861 | 2014 | 7/11/2014 | 2 |
| Native | Türkiye | Ankara | Beytepe | Ank-15 | 39.873 | 32.732 | 1038 | 2014 | 7/19/2014 | 4 |
| Native | Türkiye | Ankara | Aydın | Ank-16 | 40.166 | 32.67 | 895 | 2014 | 7/23/2014 | 3 |
| Native | Türkiye | Ankara | Üçbaş | Ank-17 | 40.375 | 32.68 | 1075 | 2014 | 7/23/2014 | 2 |
| Native | Türkiye | Ankara | Ankara | Ank-18 | 40.242 | 32.502 | 1100 | 2014 | 7/23/2014 | 2 |
| Native | Türkiye | Antalya | Bademağacı | Ant-01 | 37.206 | 30.484 | 878 | 2014 | 7/13/2014 | 2 |
| Native | Türkiye | Isparta | Burdur | Isp-01 | 37.898 | 30.351 | 916 | 2014 | 7/12/2014 | 3 |
| Native | Türkiye | Isparta | Gölbaşı | Isp-02 | 37.84 | 30.416 | 958 | 2014 | 7/14/2014 | 4 |
| Native | Türkiye | Isparta | Uluborlu | Isp-04 | 38.076 | 30.468 | 1161 | 2014 | 7/14/2014 | 3 |
| Native | Türkiye | Isparta | Taşevi | Isp-05 | 38.275 | 30.849 | 932 | 2014 | 7/14/2014 | 3 |
| Native | Türkiye | Isparta | Yalvaç | Isp-06 | 38.323 | 31.095 | 1151 | 2014 | 7/14/2014 | 3 |
| Native | Türkiye | Isparta | Yalvaç | Isp-07 | 38.323 | 31.107 | 1148 | 2014 | 7/14/2014 | 1 |
| Native | Türkiye | Isparta | Bağıllı | Isp-08 | 38.143 | 31.051 | 1023 | 2014 | 7/14/2014 | 2 |
| Native | Türkiye | Nevşehir | Göreme | Nes-02 | 38.642 | 34.826 | 1152 | 2014 | 7/17/2014 | 1 |
| Native | Türkiye | Niğde | Çamardı | Nig-01 | 37.727 | 35.018 | 1621 | 2014 | 7/17/2014 | 3 |
| Introduced | California | Marin | Petaluma | Mar-01 | 38.163 | -122.572 | 110 | 2015 | 6/11/2014 | 2 |
| Introduced | California | Contra Costa | Clayton | Ala-01 | 37.752 | -121.91 | 175 | 2015 | 6/11/2014 | 3 |
| Introduced | California | Alameda | San Ramon | Ala-02 | 37.768 | -122.008 | 292 | 2015 | 6/11/2014 | 1 |
| Introduced | California | Alameda | Fremont | Ala-03 | 37.561 | -121.861 | 202 | 2015 | 6/11/2014 | 3 |
| Introduced | California | Santa Barbara | Santa Ynez | Bar-02 | 34.547 | -119.895 | 315 | 2015 | 6/21/2015 | 3 |
| Introduced | California | Contra Costa | Martinez | Bri-08 | 37.929 | -122.152 | 284 | 2015 |  | 3 |
| Introduced | California | Butte | Oroville | But-01 | 39.536 | -121.586 | 99 | 2015 | 6/8/2014 | 2 |
| Introduced | California | Santa Clara | Milpitas | Cla-01 | 37.439 | -121.869 | 58 | 2015 | 6/12/2014 | 3 |
| Introduced | California | Santa Clara | Lexington Hills | Cla-02 | 37.194 | -121.994 | 263 | 2015 | 6/12/2014 | 4 |
| Introduced | California | Santa Clara | Gilroy | Cla-03 | 36.939 | -121.552 | 83 | 2015 | 6/12/2014 | 3 |
| Introduced | California | El Dorado | Riverton | Eld-01 | 38.773 | -120.454 | 1242 | 2015 | 6/3/2014 | 2 |
| Introduced | California | El Dorado | Kyburz | Eld-03 | 38.764 | -120.325 | 1524 | 2015 | 6/3/2014 | 2 |
| Introduced | California | El Dorado | Short Place | Eld-05 | 38.772 | -120.477 | 1219 | 2015 | 6/3/2014 | 1 |
| Introduced | California | El Dorado | Camino | Eld-06 | 38.741 | -120.663 | 950 | 2015 | 6/3/2014 | 3 |
| Introduced | California | El Dorado | Gold Hill | Eld-10 | 38.76 | -120.842 | 485 | 2015 | 6/3/2014 | 2 |
| Introduced | California | El Dorado | Folsom | Eld-13 | 38.713 | -121.063 | 242 | 2015 | 6/4/2014 | 2 |
| Introduced | California | El Dorado | Cameron Park | Eld-15 | 38.711 | -120.952 | 408 | 2015 | 6/4/2014 | 3 |
| Introduced | California | Fresno | Pinehurst | Fre-01 | 36.694 | -119.016 | 1309 | 2015 | 6/19/2015 | 3 |
| Introduced | California | Fresno | Dunlap | Fre-03 | 36.761 | -119.113 | 905 | 2015 | 6/19/2015 | 3 |
| Introduced | California | Fresno | Riverbend | Fre-04 | 36.733 | -119.49 | 126 | 2015 | 6/19/2015 | 3 |
| Introduced | California | Fresno | Fresno | Fre-06 | 36.664 | -119.719 | 94 | 2015 | 6/20/2015 | 3 |
| Introduced | California | Fresno | Coalinga | Fre-08 | 36.099 | -120.42 | 339 | 2015 | 6/20/2015 | 3 |
| Introduced | California | Fresno | Coalinga | Fre-09 | 36.105 | -120.553 | 504 | 2015 | 6/20/2015 | 4 |
| Introduced | California | Glenn | Willows | Gle-01 | 39.404 | -122.193 | 27 | 2015 | 6/7/2014 | 3 |
| Introduced | California | Glenn | Orland | Gle-02 | 39.74 | -122.202 | 80 | 2015 | 6/7/2014 | 1 |
| Introduced | California | Kern | Tehachapi | Ker-01 | 35.124 | -118.409 | 1247 | 2015 | 6/18/2015 | 3 |
| Introduced | California | Kern | Bealville | Ker-03 | 35.264 | -118.633 | 492 | 2015 | 6/18/2015 | 3 |
| Introduced | California | Kern | Lebec | Ker-04 | 34.864 | -118.882 | 1161 | 2015 | 6/18/2015 | 3 |
| Introduced | California | Kern | Frazier Springs | Ker-06 | 34.816 | -119.004 | 1705 | 2015 | 6/18/2015 | 3 |
| Introduced | California | Los Angeles | Gorman | Ker-07 | 34.765 | -118.723 | 1089 | 2015 | 6/18/2015 | 3 |
| Introduced | California | San Mateo | Woodside | Mat-01 | 37.443 | -122.265 | 169 | 2015 | 6/11/2014 | 1 |
| Introduced | California | Mendocino | Potter Valley | Men-02 | 39.321 | -123.095 | 323 | 2015 | 6/11/2014 | 1 |
| Introduced | California | Mendocino | Lakeport | Men-05 | 38.97 | -123.02 | 550 | 2015 | 6/11/2014 | 3 |
| Introduced | California | Monterey | San Lucas | Mont-01 | 36.191 | -120.754 | 735 | 2015 | 6/20/2015 | 3 |
| Introduced | California | Monterey | Jolon | Mont-02 | 36.062 | -121.185 | 449 | 2015 | 6/20/2015 | 3 |
| Introduced | California | Monterey | Bradley | Mont-03 | 35.872 | -120.87 | 229 | 2015 | 6/20/2015 | 2 |
| Introduced | California | Napa | Yountville | Nap-01 | 38.399 | -122.263 | 413 | 2015 | 6/10/2014 | 2 |
| Introduced | California | Nevada | Grass Valley | Nev-03 | 39.218 | -121.058 | 768 | 2015 | 6/5/2014 | 3 |
| Introduced | California | San Luis Obispo | San Luis Obispo | Obi-01 | 35.319 | -120.622 | 315 | 2015 | 6/20/2015 | 3 |
| Introduced | California | Placer | Monte Vista | Pla-06 | 39.187 | -120.832 | 1039 | 2015 | 6/5/2014 | 3 |
| Introduced | California | Placer | Shady Glen | Pla-07 | 39.121 | -120.941 | 735 | 2015 | 6/5/2014 | 3 |
| Introduced | California | Nevada | Applegate | Pla-11 | 39.007 | -121.013 | 607 | 2015 | 6/5/2014 | 1 |
| Introduced | California | Nevada | Auburn | Pla-13 | 38.893 | -121.084 | 363 | 2015 | 6/5/2014 | 1 |
| Introduced | California | Riverside | Lake Elsinore | Riv-01 | 33.65 | -117.364 | 477 | 2015 | 6/17/2015 | 3 |
| Introduced | California | Sacramento | Carmichael | Sac-03 | 38.611 | -121.318 | 27 | 2015 | 6/2/2014 | 1 |
| Introduced | California | Sacramento | Fair Oaks | Sac-05 | 38.638 | -121.237 | 58 | 2015 | 6/2/2014 | 2 |
| Introduced | California | California | Sacramento | Sac-11 | 38.636 | -121.502 | 4 | 2015 | 6/7/2014 | 4 |
| Introduced | California | Shasta | Redding | Sha-01 | 40.538 | -122.35 | 140 | 2015 | 6/7/2014 | 3 |
| Introduced | California | Shasta | Round Mountain | Sha-02 | 40.787 | -121.967 | 668 | 2015 | 6/7/2014 | 2 |
| Introduced | California | Shasta | Bella Vista | Sha-06 | 40.752 | -122.035 | 527 | 2015 | 6/7/2014 | 2 |
| Introduced | California | Shasta | Bethel | Sha-10 | 40.67 | -122.174 | 209 | 2015 | 6/8/2014 | 2 |
| Introduced | California | Shasta | Potem Creek | Sha-16 | 40.864 | -122.041 | 546 | 2015 | 6/8/2014 | 2 |
| Introduced | California | Shasta | Hillcrest | Sha-17 | 40.865 | -121.897 | 916 | 2015 | 6/8/2014 | 2 |
| Introduced | California | Shasta | Burney-1 | Sha-19 | 40.86 | -121.738 | 1291 | 2015 | 6/8/2014 | 3 |
| Introduced | California | Shasta | Burney-2 | Sha-20 | 40.891 | -121.651 | 963 | 2015 | 6/8/2014 | 2 |
| Introduced | California | Shasta | Hat Creek | Sha-21 | 40.82 | -121.513 | 1069 | 2015 | 6/8/2014 | 3 |
| Introduced | California | Sonoma | Calistoga | Son-01 | 38.63 | -122.67 | 199 | 2015 | 6/10/2014 | 1 |
| Introduced | California | Tehama | Red Bluff | Teh-02 | 40.347 | -121.729 | 1160 | 2015 | 6/8/2014 | 2 |
| Introduced | California | Tehama | Los Molinos | Teh-04 | 40.034 | -122.1 | 71 | 2015 | 6/8/2014 | 3 |
| Introduced | California | Tulare | Portersville | Tul-01 | 35.895 | -118.92 | 253 | 2015 | 6/19/2015 | 1 |
| Introduced | California | Ventura | Ojai | Ven-01 | 34.419 | -119.334 | 208 | 2015 | 6/21/2015 | 1 |
| Introduced | California | Ventura | Casitas Springs | Ven-02 | 34.374 | -119.306 | 206 | 2015 | 6/21/2015 | 1 |
| Introduced | California | Yolo | Woodland | Yol-01 | 38.699 | -121.765 | 17 | 2015 | 6/7/2014 | 2 |
| Introduced | Idaho | Idaho | White Bird | Ida-01 | 45.665 | -116.295 | 600 | 2015 | 7/9/2015 | 2 |
| Introduced | Idaho | Lewis | Craigmont | Lew-01 | 46.202 | -116.416 | 1100 | 2015 | 7/10/2015 | 3 |
| Introduced | Idaho | Nez Perce | Lapwai Creek | Lew-02 | 46.358 | -116.788 | 556 | 2015 | 7/10/2015 | 2 |
| Introduced | Idaho | Nez Perce | Lenore | Lew-03 | 46.512 | -116.547 | 474 | 2015 | 7/10/2015 | 3 |
| Introduced | Idaho | Idaho | Sevenmile Creek | Lew-04 | 46.171 | -116.074 | 748 | 2015 | 7/10/2015 | 3 |
| Introduced | Oregon | Umatilla | Pendleton | Ar | 45.696 | -118.871 | 438 | 2012 | 8/13/2012 | 3 |
| Introduced | Oregon | Union | La Grande | Cd | 45.374 | -117.829 | 903 | 2012 | 8/14/2012 | 3 |
| Introduced | Oregon | Umatilla | Milton Freewater | Ldc | 45.909 | -118.415 | 423 | 2012 | 8/16/2012 | 3 |
| Introduced | Oregon | Umatilla | Pendleton | Mcr | 45.603 | -118.8 | 397 | 2012 | 8/13/2012 | 3 |
| Introduced | Oregon | Grant | John Day | Wrc | 44.45 | -119.391 | 882 | 2012 | 8/12/2012 | 2 |
| Introduced | Washington | Franklin | Basin City | Bc | 46.594 | -119.159 | 205 | 2012 | 8/16/2012 | 3 |
| Introduced | Washington | Asotin | Asotin | Cr | 46.321 | -117.118 | 478 | 2012 | 8/15/2012 | 4 |
| Introduced | Washington | Klickitat | White Salmon | Crb | 45.701 | -121.411 | 166 | 2012 | 8/17/2012 | 5 |
| Introduced | Washington | Columbia | Dayton | Dhr | 46.333 | -117.823 | 816 | 2012 | 8/15/2012 | 2 |
| Introduced | Washington | Walla Walla | Prescott | Rr | 46.272 | -118.349 | 363 | 2012 | 8/16/2012 | 2 |
| Introduced | Washington | Klickitat | Maryhill | Shm | 45.694 | -120.807 | 176 | 2012 | 8/17/2012 | 5 |
| Introduced | Washington | Walla Walla | Burbank | We | 46.268 | -118.777 | 254 | 2012 | 8/16/2012 | 2 |
| Introduced | Washington | Walla Walla | Walla Walla | Wwb | 46.074 | -118.287 | 335 | 2012 | 8/15/2012 | 1 |

**Table S2.** Presence records for *Eustenopus villosus* from its native range derived from the literature, museum records, personal communications, and this study. The country, province, locality, latitude, longitude, date of collection (if known), and reference are indicated for each record. Geographic coordinates were estimated from city names for records lacking this information (Estimated = 1).

| **Country** | **Province** | **Locality** | **Latitude** | **Longitude** | **Estimated** | **Date** | **Ref** |
| --- | --- | --- | --- | --- | --- | --- | --- |
| Bulgaria | Blagoevgrad | Petrich | 41.4 | 23.21 | 1 |  | 1 |
| Bulgaria | Burgas | Burgas | 42.532 | 27.471 | 0 | 6/22/2022 | 2 |
| Bulgaria | Plovdiv | Plovdiv | 42.135 | 24.745 | 1 | 6/26/2014 | 3 |
| Georgia | Mengrelia | Unknown | 42.492 | 41.863 | 1 |  | 4 |
| Greece | Aetoliua-Acarnania | Nafpaktos | 38.423 | 21.748 | 0 | 6/30/2014 | 5 |
| Greece | Aetoliua-Acarnania | Thermo | 38.481 | 21.639 | 0 | 6/30/2014 | 5 |
| Greece | Aetoliua-Acarnania | Agrinio | 38.618 | 21.423 | 0 | 6/30/2014 | 5 |
| Greece | Kilkis | Vafiochori | 41.015 | 22.67 | 0 | 7/3/2014 | 5 |
| Greece | Kilkis | Cherso | 41.089 | 22.798 | 0 | 7/3/2014 | 5 |
| Greece | Kilkis | Dorsato | 41.123 | 22.795 | 0 | 7/3/2014 | 5 |
| Greece | Kilkis | Doirani | 41.187 | 22.758 | 0 |  | 9 |
| Greece | Lesbos | Weiden 1km W. Sikaminea | 39.35 | 26.283 | 0 | 3/6/2016 | 6 |
| Greece | Pella | Pella | 40.827 | 22.444 | 0 | 7/3/2014 | 5 |
| Greece | Pella | Stathis | 40.952 | 22.502 | 0 | 7/3/2014 | 5 |
| Greece | Pierra | Pierra | 40.262 | 22.436 | 0 | 7/5/2014 | 5 |
| Greece | Serres | Alistrati | 41.064 | 23.959 | 1 | 5/23/2016 | 7 |
| Greece | Thessaloniki | Drimos | 40.772 | 22.949 | 0 | 7/3/2014 | 5 |
| Greece | Thessaloniki | Oreokastro | 40.726 | 22.928 | 0 | 7/3/2014 | 5 |
| Greece | Thessaloniki | Lagkadikia | 40.633 | 23.238 | 0 | 7/4/2014 | 5 |
| Greece | Thessaloniki | Thessaloniki | 40.581 | 22.991 | 0 |  | 8 |
| Greece | Thessaloniki | Thermi | 40.548 | 23.019 | 1 |  | 8 |
| Greece | Thessaloniki | Oreokastro | 40.727 | 22.92 | 0 |  | 8 |
| Greece | Thessaloniki | Kilkis | 41.221 | 22.75 | 0 |  | 8 |
| Iran | Golestan | Kordkoy | 36.791 | 54.106 | 0 |  | 10 |
| Iran | Khorasan | Unknown | 35.73 | 59.25 | 0 |  | 11 |
| Moldova | Ștefan Vodă District | Pananca | 46.408 | 30.096 | 0 | 5/30/1952 | 12 |
| Türkiye | Adana | Pozantı | 37.378 | 34.843 | 0 | 7/15/2014 | 5 |
| Türkiye | Adana | Kadirli | 37.369 | 36.1 | 1 | 5/11/1993 | 5 |
| Türkiye | Adana | Ponzantı A | 37.515 | 34.939 | 0 |  | 13 |
| Türkiye | Adana | Ponzantı B | 37.737 | 35.015 | 0 |  | 13 |
| Türkiye | Adana | Pozantı | 37.516 | 34.939 | 0 |  | 13 |
| Türkiye | Afyon | Bolvadin | 38.757 | 31.047 | 0 | 7/12/2014 | 5 |
| Türkiye | Aksaray | 50 km E of Aksaray | 38.545 | 34.491 | 0 | 6/17/2003 | 14 |
| Türkiye | Aksaray | 8-10 km E of Aksaray | 38.416 | 34.053 | 0 | 7/22/2009 | 14 |
| Türkiye | Ankara | Akyurt | 40.079 | 33.012 | 0 | 7/10/2014 | 5 |
| Türkiye | Ankara | Dağdemir | 40.23 | 33.458 | 0 | 7/10/2014 | 5 |
| Türkiye | Ankara | Kalecik | 40.121 | 33.429 | 0 | 7/10/2014 | 5 |
| Türkiye | Ankara | Keskin | 39.708 | 33.576 | 0 | 7/10/2014 | 5 |
| Türkiye | Ankara | Yenikent | 40.008 | 32.524 | 0 | 7/11/2014 | 5 |
| Türkiye | Ankara | Ayaş | 40.032 | 32.285 | 0 | 7/11/2014 | 5 |
| Türkiye | Ankara | Beypazarı | 40.129 | 32.001 | 0 | 7/11/2014 | 5 |
| Türkiye | Ankara | Uruş | 40.296 | 32.093 | 0 | 7/11/2014 | 5 |
| Türkiye | Ankara | Uruş | 40.288 | 32.116 | 0 | 7/11/2014 | 5 |
| Türkiye | Ankara | Uruş | 40.256 | 32.133 | 0 | 7/11/2014 | 5 |
| Türkiye | Ankara | Beytepe | 39.873 | 32.732 | 0 | 7/19/2014 | 5 |
| Türkiye | Ankara | Aydın | 40.166 | 32.67 | 0 | 7/23/2014 | 5 |
| Türkiye | Ankara | Üçbaş | 40.375 | 32.68 | 0 | 7/23/2014 | 5 |
| Türkiye | Ankara | Ankara | 40.242 | 32.502 | 0 | 7/23/2014 | 5 |
| Türkiye | Ankara | Beytepe Campus area | 39.867 | 32.733 | 1 | 6/30/1989 | 5 |
| Türkiye | Ankara | METU Campus area | 39.876 | 32.763 | 1 | 5/27/1992 | 5 |
| Türkiye | Ankara | Beypazarı | 40.167 | 31.921 | 1 | 6/11/1995 | 5 |
| Türkiye | Ankara | 18 km NW of Şereflikoçhisar, Tuz lake environ | 39.092 | 33.393 | 0 | 6/21/2005 | 14 |
| Türkiye | Ankara | 18.6 km SE of Şereflikoçhisar, Tuz lake environ | 38.814 | 33.604 | 0 | 6/23/2005 | 14 |
| Türkiye | Antalya | Bademağacı | 37.206 | 30.484 | 0 | 7/13/2014 | 5 |
| Türkiye | Elazığ | 23.4 km NE of Kovancılar | 38.88 | 40.021 | 0 | 7/3/2005 | 15 |
| Türkiye | Eskişehir | Sarıcakaya | 40.038 | 30.618 | 1 | 6/26/1989 | 5 |
| Türkiye | Isparta | Uluborlu | 38.076 | 30.468 | 0 | 7/14/2014 | 5 |
| Türkiye | Isparta | Taşevi | 38.275 | 30.849 | 0 | 7/14/2014 | 5 |
| Türkiye | Isparta | Bağıllı | 38.143 | 31.051 | 0 | 7/14/2014 | 5 |
| Türkiye | Isparta | Burdur | 37.898 | 30.351 | 0 | 7/14/2014 | 5 |
| Türkiye | Isparta | Gölbaşı | 37.84 | 30.416 | 0 | 7/14/2014 | 5 |
| Türkiye | Isparta | Yalvaç | 38.322 | 31.094 | 0 | 7/14/2014 | 5 |
| Türkiye | Isparta | Eğirdir | 38.023 | 30.872 | 1 | 6/30/1992 | 5 |
| Türkiye | Isparta | Yalvaç | 38.295 | 31.18 | 1 | 7/1/1992 | 5 |
| Türkiye | Isparta | Gelendost | 38.123 | 31.011 | 1 | 7/1/1992 | 5 |
| Türkiye | Isparta | Uluborlu | 38.083 | 30.45 | 1 | 7/2/1992 | 5 |
| Türkiye | Isparta | Keçiborlu | 37.942 | 30.302 | 1 | 6/2/1992 | 5 |
| Türkiye | Kayseri | İncesu | 38.622 | 35.184 | 1 | 6/13/1990 | 5 |
| Türkiye | Kayseri | 18 km W of Kayseri | 38.729 | 35.272 | 1 | 6/9/2004 | 14 |
| Türkiye | Kayseri | 22 km W of Kayseri | 38.729 | 35.226 | 1 | 6/23/2004 | 14 |
| Türkiye | Kayseri | 3 km N of İncesu | 38.646 | 35.185 | 1 | 6/23/2004 | 14 |
| Türkiye | Kayseri | Kayseri | 38.721 | 35.483 | 1 | 6/29/1980 | 16 |
| Türkiye | Kayseri | 18 km west of Kayseri | 38.749 | 35.295 | 1 | 6/9/2004 | 17 |
| Türkiye | Kırıkkale | 20 km E of Kırıkkale | 39.924 | 33.376 | 0 | 6/20/2005 | 14 |
| Türkiye | Kırıkkale | 11 km W of Kırıkkale | 39.928 | 33.404 | 0 | 6/20/2005 | 14 |
| Türkiye | Mardin | Mardin Dağları, Hwy 380 | 37.447 | 41.029 | 0 | 6/10/2006 | 15 |
| Türkiye | Nevşehir | Gülşehir | 38.743 | 34.626 | 1 | 5/19/1989 | 5 |
| Türkiye | Nevşehir | 9 km NE of Ürgüp | 38.681 | 34.989 | 1 | 6/9/2004 | 14 |
| Türkiye | Nevşehir | 18 km E of Avanos | 38.712 | 34.64 | 1 | 6/23/2004 | 14 |
| Türkiye | Nevşehir | 7.5 km NW of Avanos | 38.751 | 34.776 | 1 | 6/23/2004 | 14 |
| Türkiye | Nevşehir | Avanos environ | 38.715 | 34.846 | 1 | 6/23/2004 | 14 |
| Türkiye | Nevşehir | Göreme | 38.645 | 34.829 | 0 |  | 18 |
| Türkiye | Nevşehir | Near Göreme | 38.665 | 34.818 | 0 |  | 19 |
| Türkiye | Nevşehir | Göreme | 38.642 | 34.826 | 0 | 7/17/2014 | 5 |
| Türkiye | Niğde | Çamardı | 37.833 | 34.987 | 0 |  | 18 |
| Türkiye | Niğde | Çamardı | 37.727 | 35.018 | 0 | 7/17/2014 | 5 |
| Türkiye | Şanlıurfa | Halfeti Road | 37.236 | 37.927 | 0 | 6/8/2006 | 15 |
| Ukraine | Crimea | Yedi Quyu, Voykove [=Kidirlez] | 45.387 | 36.433 | 0 | 6/4/1952 | 20 |
| Ukraine | Crimea | Nyzhniohirskyi | 45.443 | 34.743 | 0 | 6/18/1951 | 20 |
| Ukraine | Crimea | Feodosiya | 45.026 | 35.374 | 0 | 6/10/1951 | 20 |
| Ukraine | Crimea | Feodosia | 45.035 | 35.369 | 0 |  | 21 |
| Ukraine | Crimea | Nizhnegorskiy Distr. | 45.446 | 34.745 | 0 |  | 21 |
| Ukraine | Crimea | Yedi Quyu Distr., Voykove | 45.325 | 36.085 | 0 |  | 21 |
| Ukraine | Odessa | Bilyayivka, Mayaky | 46.421 | 30.262 | 0 | 7/19/1951 | 20 |
| Ukraine | Odessa | Bylyaivka Distr., Mayaky | 46.414 | 30.262 | 0 |  | 21 |

**References**

1. Angelov, P. (1978). *Fauna of Bulgaria; vol. 7; Coleoptera, Curculionidae, Part II: Brachyderinae, Brachycerinae, Tanymecinae, Cleoninae, Curculioninae, Myorhininae.* Sofia: Bulgarian Academy of Sciences.
2. Mikat78. (2022). iNaturalist observation. <https://www.inaturalist.org/observations/123207744> (accessed September 8, 2025).
3. Di Cristina, F. (2014). Field trip in Serbia and Bulgaria. <http://www.bbcaonlus.org/2014> (accessed September 8, 2025).
4. Cholokava, A. (2008). The weevil beetles (Rhynchitidae, Attelabidae, Apionidae, Nanophyidae, Dryophthoridae, Curculionidae) of Georgia. *Proceedings of the Institute of Zoology*, *23*, 77–123. <https://dspace.nplg.gov.ge/bitstream/1234/102378/1/Shromebi_2008_N23.pdf>.
5. This study.
6. Winkelmann, H., Bahr, F., & Messutat, J. (2021). First supplement to the checklist of the weevil fauna (Coleoptera, Curculionoidea) of Lesbos Island, Greece. *Parnassiana Archives*, *9*, 23–39. <https://www.orion-berlin.de/verein/bibl/winkelmann/Winkelmann_2021.pdf> (accessed September 8, 2025).
7. Winkelmann, H., & Bahr, F. (2022). Neue Rüsselkäferfunde (Coleoptera: Curculionoidea) aus Nordgriechenland und Ergänzungen zur Jahresexkursion 2016 des Curculio-Institutes Neue Rüsselkäferfunde aus Nordgriechenland. *WeevilNews*, *100*, 1–28. <https://curci.de/data/weevilnews/weevilnews_100.pdf> (accessed September 8, 2025).
8. Kashefi, J. (2013). Personal communication, February 12, 2013.
9. Fornasari, L., Turner, C. E., & Andres, L. A. (1991). *Eustenopus villosus* (Coleoptera: Curculionidae) for biological control of yellow starthistle (Asteraceae: Cardueae) in North America. *Environmental Entomology*, *20*(4), 1187–1194. <https://doi.org/10.1093/ee/20.4.1187>.
10. Ghahari, H., Arzanov, Y. G., Legalov, A. A., Tabari, M., & Ostovan, H. (2010). Weevils (Coleoptera: Curculionidae) from Iranian rice fields and surrounding grasslands. *Munis Entomology & Zoology Journal*, *5*, 163–169. <https://www.munisentzool.org/Issue/abstract/weevils-coleoptera-curculionidae-from-iranian-rice-fields-and-surrounding-grasslands_239>.
11. Legalov, A. A., Ghahari, H., & Arzanov, Y. G. (2010). Annotated catalogue of Curculionid-beetles (Coleoptera: Anthribidae, Rhynchitidae, Attelabidae, Brentidae, Brachyceridae, Dryophthoridae and Curculionidae) of Iran. *Amurian Zoological Journal*, *2*, 191–244. <https://doi.org/10.33910/1999-4079-2010-2-3-191-244>.
12. Volovnik, S. (2020). Personal communication, July 10, 2020.
13. Uygur, S., Smith, L., Uygur, F. N., & Türkiye, A. (2012). The effects of seed feeding insects on seed production of yellow starthistle (*Centaurea solstitialis* L.) in Adana province in southern Türkiye. *Türkiye Biyolojik Mücadele Dergisi*, *3*, 99–120. <https://dergipark.org.tr/tr/pub/tbmd/article/240143>.
14. Gultekin, L. (2014). Personal communication, March 17, 2014.
15. Korotyaev, B. A., Gültekin, L., Volkovitsh, M. G., Dorofeyev, V. I., & Konstantinov, A. S. (2016). Bioindicator beetles and plants in desertified and eroded lands in Türkiye. *Journal of Insect Biodiversity*, *4*, 1–47. <https://www.mapress.com/jib/article/view/2016.4.1>.
16. Pehlivan, E., Karsavuran, Y., & Tezcan, S. (2005). Contributions to the knowledge of the Lixinae (Coleoptera: Curculionidae) from Türkiye. *Türkiye Biyolojik Mücadele Dergisi*, *29*, 259–272. <https://dergipark.org.tr/tr/pub/entoted/article/75937>.
17. Gültekin, L., Cristofaro, M., Tronci, C., & Smith, L. (2008). Natural history studies for the preliminary evaluation of *Larinus filiformis* (Coleoptera: Curculionidae) as a prospective biological control agent of yellow starthistle. *Environmental Entomology*, *37*(5), 1185–1199. <https://doi.org/10.1093/ee/37.5.1185>.
18. Uygur, S., Smith, L., Uygur, F. N., Cristofaro, M., & Balciunas, J. (2004). Population densities of yellow starthistle (*Centaurea solstitialis*) in Türkiye. *Weed Science*, *52*, 746–753. <https://doi.org/10.1614/WS-03-150R1>.
19. Cristafaro, M. (2024). Personal communication, March 19, 2024.
20. Ukrainian Biodiversity Information Network. (2025). *Eustenopus villosus* (Boheman, 1843). <https://www.ukrbin.com/index.php?id=15526> (accessed September 8, 2025).
21. Yunakov, N., Nazarenko, V., Filimonov, R., & Volovnik, S. (2018). A survey of the weevils of Ukraine (Coleoptera: Curculionoidea). *Zootaxa*, *4404*, 1–494. <https://www.mapress.com/zt/article/view/23233>.

**Table S3.** Summary of loci associated with environmental predictors in native and introduced populations of *Eustenopus villosus.* The number of loci identified for each predictor as well as the total and average number identified across all five predictors is indicated for the redundancy analysis (RDA), partial redundancy analysis (pRDA), Bayenv, latent factor mixed models (LFMM), Gradient Forest (GF), and GF using standardized allele frequencies from Bayenv (GF-X). Environmental predictors include: bio2 = mean diurnal temperature range (mean of monthly max temperature − min temperature), bio5 = maximum temperature of the warmest month, bio6 = minimum temperature of the coldest month, bio17 = precipitation of the driest quarter, and bio19 = precipitation of the coldest quarter. Note that the total number of candidate SNPs may not be equal to the sum of the SNPs associated with the environmental predictors because SNPs may be strongly associated with more than one predictor (e.g., in the RDA, a SNP can be classified as a candidate on more than one axis).

|  |  | Environmental predictors | | | | |  |  |
| --- | --- | --- | --- | --- | --- | --- | --- | --- |
| Method | Range | bio2 | bio5 | bio6 | bio17 | bio19 | Total | Average |
| RDA | Native | 30 | 20 | 214 | 59 | 32 | 352 | 71 |
|  | Introduced | 78 | 49 | 63 | 74 | 78 | 329 | 68.4 |
| pRDA | Native | 71 | 72 | 79 | 84 | 50 | 332 | 71.2 |
|  | Introduced | 76 | 90 | 35 | 66 | 90 | 345 | 71.4 |
| Bayenv | Native | 0 | 20 | 0 | 116 | 0 | 136 | 25.2 |
|  | Introduced | 0 | 102 | 0 | 71 | 0 | 173 | 34.6 |
| LFMM | Native | 2 | 14 | 22 | 0 | 20 | 45 | 11.6 |
|  | Introduced | 5 | 0 | 0 | 0 | 1 | 6 | 1.2 |
| GF | Native | 23 | 34 | 148 | 48 | 81 | 334 | 66.8 |
|  | Introduced | 75 | 16 | 21 | 13 | 17 | 142 | 28.4 |
| GF-X | Native | 2 | 42 | 10 | 2 | 28 | 84 | 16.8 |
|  | Introduced | 0 | 0 | 0 | 2 | 0 | 2 | 0.4 |

**Table S4.** Population genomic variation in *Eustenopus villosus*. Observed heterozygosity (H_O_), expected heterozygosity (H_E_), inbreeding coefficient (F_IS_), per-site nucleotide diversity (π), and allelic richness (A_R_) are shown for (a) non-candidate loci, (b) candidate loci in the native range, and (c) candidate loci in the introduced range. Groupings include the native geographic regions (Greece and Türkiye) and serial founding events in the introduced range. Founding classification: P = primary, P/S = primary/secondary, S/T = secondary/tertiary, S/T/Q = secondary/tertiary/quaternary, T/Q = tertiary/quaternary.

(a) Non-candidate loci

| **Grouping** | **History** | **H_O_** | **H_O_ SE** | **H_E_** | **H_E_ SE** | **F_IS_** | **F_IS_ SE** | **π** | **π (SE)** | **A_R_** | **A_R_ SE** |
| --- | --- | --- | --- | --- | --- | --- | --- | --- | --- | --- | --- |
| Türkiye | Türkiye | 0.1424 | 0.0019 | 0.1627 | 0.0021 | 0.1247 | 0.0028 | 1.1625 | 0.0021 | 0.1625 | 0.0021 |
| Greece | Greece | 0.1474 | 0.0020 | 0.1615 | 0.0020 | 0.0876 | 0.0031 | 1.1613 | 0.0020 | 0.1613 | 0.0019 |
| F1 | S/T/Q | 0.1428 | 0.0024 | 0.1554 | 0.0023 | 0.0807 | 0.0041 | 1.1543 | 0.0023 | 0.1543 | 0.0022 |
| OR | S/T/Q | 0.1359 | 0.0019 | 0.1603 | 0.0021 | 0.1359 | 0.0035 | 1.1586 | 0.0021 | 0.1722 | 0.0021 |
| I2 | T/Q | 0.1536 | 0.0023 | 0.1651 | 0.0022 | 0.0697 | 0.0038 | 1.1643 | 0.0022 | 0.1643 | 0.0022 |
| WA | T/Q | 0.1276 | 0.0018 | 0.1592 | 0.0021 | 0.1276 | 0.0037 | 1.1527 | 0.0021 | 0.1704 | 0.0021 |
| E | S/T | 0.1454 | 0.0022 | 0.1587 | 0.0022 | 0.0840 | 0.0036 | 1.1579 | 0.0021 | 0.1579 | 0.0021 |
| D | S/T | 0.1448 | 0.0023 | 0.1558 | 0.0022 | 0.0703 | 0.0038 | 1.1550 | 0.0022 | 0.1550 | 0.0021 |
| F2 | S/T/Q | 0.1483 | 0.0022 | 0.1595 | 0.0022 | 0.0703 | 0.0037 | 1.1588 | 0.0022 | 0.1588 | 0.0021 |
| P | P | 0.1426 | 0.0019 | 0.1586 | 0.0020 | 0.1010 | 0.0031 | 1.1583 | 0.0020 | 0.1583 | 0.0019 |
| B | P/S | 0.1385 | 0.0026 | 0.1551 | 0.0025 | 0.1071 | 0.0047 | 1.1523 | 0.0025 | 0.1523 | 0.0024 |
| C | S/T | 0.1405 | 0.0022 | 0.1552 | 0.0022 | 0.0944 | 0.0039 | 1.1543 | 0.0021 | 0.1543 | 0.0021 |
| H | T/Q | 0.1421 | 0.0022 | 0.1551 | 0.0022 | 0.0840 | 0.0039 | 1.1544 | 0.0021 | 0.1544 | 0.0021 |
| G2 | T/Q | 0.1241 | 0.0021 | 0.1514 | 0.0021 | 0.1798 | 0.0036 | 1.1485 | 0.0023 | 0.1485 | 0.0023 |
| ID | S/T | 0.1426 | 0.0022 | 0.1585 | 0.0024 | 0.1008 | 0.0045 | 1.1578 | 0.0021 | 0.1578 | 0.0021 |
| I1 | T/Q | 0.1478 | 0.0021 | 0.1570 | 0.0021 | 0.0588 | 0.0032 | 1.1566 | 0.0021 | 0.1566 | 0.0020 |
| G1 | T/Q | 0.1490 | 0.0022 | 0.1587 | 0.0021 | 0.0614 | 0.0036 | 1.1582 | 0.0021 | 0.1582 | 0.0021 |
| A | P/S | 0.1475 | 0.0023 | 0.1589 | 0.0022 | 0.0719 | 0.0040 | 1.1581 | 0.0022 | 0.1581 | 0.0022 |

(b) Candidate loci (native range)

| **Grouping** | **History** | **H_O_** | **H_O_ SE** | **H_E_** | **H_E_ SE** | **F_IS_** | **F_IS_ SE** | **π** | **π (SE)** | **A_R_** | **A_R_ SE** |
| --- | --- | --- | --- | --- | --- | --- | --- | --- | --- | --- | --- |
| Türkiye | Türkiye | 0.3343 | 0.0069 | 0.4015 | 0.0064 | 0.1674 | 0.0116 | 0.4009 | 0.0064 | 1.4009 | 0.0064 |
| Greece | Greece | 0.3319 | 0.0081 | 0.3974 | 0.0069 | 0.1648 | 0.0141 | 0.3961 | 0.0069 | 1.3961 | 0.0069 |
| F1 | S/T/Q | 0.3278 | 0.0126 | 0.3698 | 0.0102 | 0.1136 | 0.0223 | 0.3658 | 0.0099 | 1.3658 | 0.0099 |
| OR | S/T/Q | 0.3133 | 0.0090 | 0.3765 | 0.0085 | 0.1679 | 0.0157 | 0.3746 | 0.0085 | 1.3746 | 0.0085 |
| I2 | T/Q | 0.3403 | 0.0114 | 0.3756 | 0.0095 | 0.0940 | 0.0195 | 0.3732 | 0.0094 | 1.3732 | 0.0094 |
| WA | T/Q | 0.2783 | 0.0088 | 0.3697 | 0.0084 | 0.2367 | 0.0162 | 0.3668 | 0.0083 | 1.3668 | 0.0083 |
| E | S/T | 0.3311 | 0.0112 | 0.3765 | 0.0089 | 0.1205 | 0.0198 | 0.3738 | 0.0088 | 1.3738 | 0.0088 |
| D | S/T | 0.3339 | 0.0118 | 0.3702 | 0.0095 | 0.0981 | 0.0204 | 0.3678 | 0.0094 | 1.3678 | 0.0094 |
| F2 | S/T/Q | 0.3413 | 0.0114 | 0.3771 | 0.0092 | 0.0949 | 0.0195 | 0.3749 | 0.0091 | 1.3749 | 0.0091 |
| P | P | 0.3224 | 0.0086 | 0.3767 | 0.0076 | 0.1442 | 0.0158 | 0.3758 | 0.0076 | 1.3758 | 0.0076 |
| B | P/S | 0.3178 | 0.0148 | 0.3597 | 0.0123 | 0.1164 | 0.0262 | 0.3530 | 0.0117 | 1.3530 | 0.0117 |
| C | S/T | 0.3258 | 0.0114 | 0.3732 | 0.0091 | 0.1270 | 0.0209 | 0.3704 | 0.0090 | 1.3704 | 0.0090 |
| H | T/Q | 0.3229 | 0.0114 | 0.3739 | 0.0091 | 0.1365 | 0.0209 | 0.3714 | 0.0085 | 1.3714 | 0.0085 |
| G2 | T/Q | 0.2875 | 0.0102 | 0.3526 | 0.0086 | 0.1848 | 0.0188 | 0.3449 | 0.0105 | 1.3449 | 0.0105 |
| ID | S/T | 0.3178 | 0.0116 | 0.3705 | 0.0109 | 0.1423 | 0.0211 | 0.3682 | 0.0086 | 1.3682 | 0.0086 |
| I1 | T/Q | 0.3390 | 0.0104 | 0.3670 | 0.0088 | 0.0765 | 0.0163 | 0.3661 | 0.0088 | 1.3661 | 0.0088 |
| G1 | T/Q | 0.3292 | 0.0111 | 0.3658 | 0.0090 | 0.1001 | 0.0195 | 0.3640 | 0.0089 | 1.3640 | 0.0089 |
| A | P/S | 0.3462 | 0.0123 | 0.3811 | 0.0092 | 0.0917 | 0.0222 | 1.3785 | 0.0091 | 0.3785 | 0.0091 |

(c) Candidate loci (introduced range)

| **Grouping** | **History** | **H_O_** | **H_O_ SE** | **H_E_** | **H_E_ SE** | **F_IS_** | **F_IS_ SE** | **π** | **π (SE)** | **A_R_** | **A_R_ SE** |
| --- | --- | --- | --- | --- | --- | --- | --- | --- | --- | --- | --- |
| Türkiye | Türkiye | 0.2902 | 0.0085 | 0.3362 | 0.0086 | 0.1368 | 0.0116 | 0.3990 | 0.0086 | 1.3358 | 0.0086 |
| Greece | Greece | 0.3599 | 0.0083 | 0.3998 | 0.0065 | 0.0998 | 0.0141 | 0.4065 | 0.0065 | 1.3990 | 0.0065 |
| F1 | S/T/Q | 0.3440 | 0.0118 | 0.3996 | 0.0086 | 0.1391 | 0.0223 | 0.4067 | 0.0084 | 1.3948 | 0.0084 |
| OR | S/T/Q | 0.3458 | 0.0081 | 0.4216 | 0.0059 | 0.1799 | 0.0157 | 0.4193 | 0.0059 | 1.4193 | 0.0059 |
| I2 | T/Q | 0.3789 | 0.0112 | 0.4128 | 0.0077 | 0.0820 | 0.0195 | 0.4159 | 0.0077 | 1.4104 | 0.0077 |
| WA | T/Q | 0.3215 | 0.0079 | 0.4120 | 0.0060 | 0.2198 | 0.0162 | 0.4091 | 0.0059 | 1.4091 | 0.0059 |
| E | S/T | 0.3659 | 0.0105 | 0.4193 | 0.0071 | 0.1274 | 0.0198 | 0.4019 | 0.0070 | 1.4159 | 0.0070 |
| D | S/T | 0.3567 | 0.0101 | 0.4061 | 0.0077 | 0.1217 | 0.0204 | 0.4109 | 0.0076 | 1.4030 | 0.0076 |
| F2 | S/T/Q | 0.3709 | 0.0110 | 0.4172 | 0.0071 | 0.1111 | 0.0195 | 0.3948 | 0.0070 | 1.4144 | 0.0070 |
| P | P | 0.3523 | 0.0082 | 0.4182 | 0.0057 | 0.1577 | 0.0158 | 0.4204 | 0.0056 | 1.4171 | 0.0056 |
| B | P/S | 0.3641 | 0.0142 | 0.4116 | 0.0106 | 0.1156 | 0.0262 | 0.4144 | 0.0102 | 1.4065 | 0.0102 |
| C | S/T | 0.3481 | 0.0110 | 0.4056 | 0.0075 | 0.1418 | 0.0209 | 0.4017 | 0.0074 | 1.4019 | 0.0074 |
| H | T/Q | 0.3458 | 0.0110 | 0.4002 | 0.0075 | 0.1358 | 0.0209 | 0.4015 | 0.0072 | 1.3975 | 0.0072 |
| G2 | T/Q | 0.3150 | 0.0103 | 0.3660 | 0.0072 | 0.1394 | 0.0188 | 0.3975 | 0.0098 | 1.3605 | 0.0098 |
| ID | S/T | 0.3402 | 0.0117 | 0.4143 | 0.0100 | 0.1787 | 0.0211 | 0.3605 | 0.0072 | 1.4109 | 0.0072 |
| I1 | T/Q | 0.3710 | 0.0094 | 0.4031 | 0.0073 | 0.0795 | 0.0163 | 0.4017 | 0.0073 | 1.4017 | 0.0073 |
| G1 | T/Q | 0.3476 | 0.0103 | 0.4004 | 0.0076 | 0.1319 | 0.0195 | 0.3977 | 0.0075 | 1.3977 | 0.0075 |
| A | P/S | 0.3616 | 0.0119 | 0.4101 | 0.0077 | 0.1182 | 0.0222 | 0.3358 | 0.0076 | 1.4067 | 0.0076 |

**Figure S1.** Geographic origins of records for *Eustenopus villosus* used for this study. The origin for all introduced populations in the western United States (two sites near Thessaloniki, Greece), primary release sites, sampling sites for genetic material, and sites used to classify the climatic niche for the native and introduced ranges are indicated.

**Figure S2.** Boxplots of the inbreeding coefficient (*F*_IS_) based on RAD loci in *Eustenopus villosus* individuals at different -*M* and -*n* values in STACKS, where -*M* is the number of nucleotide differences allowed between loci when processing a single individual and -*n* is the number of nucleotide differences allowed between loci when creating the catalog of loci. Boxplots show median (horizontal line), interquartile range (box), and minimum and maximum values (whiskers). Positive values for *F*_IS_ may indicate excess homozygosity due to failure to cluster loci under restrictive mismatch values, whereas negative values for *F*_IS_ may indicate excess heterozygosity due to clustering of paralogous loci.

**Figure S3.** Estimated introduction history and groupings of the sampled *Eustenopus villosus* populations in the western United States. Groups were classified by introduction history (P = primary, P/S = primary/secondary, S/T = secondary/tertiary, S/T/Q = secondary/tertiary/quaternary, T/Q = tertiary/quaternary). Populations in Washington were excluded from population genomic analyses owing to an absence of release records.

**Figure S4.** Plot of cross-validation errors at different values of K (number of populations) according to ADMIXTURE analyses of single nucleotide polymorphism data for *Eustenopus villosus*.

**Figure S5**. Map with pie charts depicting the geographic distribution of three genetic clusters in introduced populations of *Eustenopus villosus* in the western United States. Pie slices indicate the proportion of each cluster. Pie sizes are proportional to the number of samples in the population.

| **(a) GF: Native**   | **(b) GF-X: Native**   |
| --- | --- |
| **(c) GF: Introduced**   | **(d) GF-X: Introduced**   |

**Figure S6**. Results of the gradient forest analysis on raw allele frequencies (GF) and standardized allele frequencies (GF-X) for native (a, b) and introduced (c, d) populations of *Eustenopus villosus*. The accuracy importance and R²-weighted importance of environmental predictors that explain allele frequencies are indicated for each analysis. Predictors include five bioclimatic variables: bio2 = mean diurnal temperature range (mean of monthly max temperature − min temperature), bio5 = maximum temperature of the warmest month, bio6 = minimum temperature of the coldest month, bio17 = precipitation of the driest quarter, and bio19 = precipitation of the coldest quarter.
